# SIRT2 antagonizes MOF function during mitotic entry

**DOI:** 10.1126/sciadv.aeb2915

**Published:** 2026-06-19

**Authors:** María Espinosa-Alcantud, Núria Sima, Irene Fernández-Duran, Anna Marazuela-Duque, Gerard Martínez-Cebrián, Anna Guitart-Solanes, Andrés Gámez-García, Paloma Martínez-Redondo, Laia Bosch-Presegué, Berta N. Vazquez, Luis Paños, Joan Josep Bech, Tim Thomas, Marian A. Martinez-Balbás, Anne K. Voss, Marcus Krüger, Lourdes Serrano, Mireia Olivella, Sergi Cuartero, Carolina de la Torre, Alejandro Vaquero

**Affiliations:** ^1^ Chromatin Biology Laboratory, Cancer Epigenetics and Biology Program (PEBC), Bellvitge Biomedical Research Institute (IDIBELL), L’Hospitalet de Llobregat, Barcelona, Spain.; ^2^ Chromatin Biology Laboratory, Josep Carreras Leukaemia Research Institute (IJC), Badalona, Spain.; ^3^ Transcriptional Dynamics in Leukemia Laboratory, Josep Carreras Leukaemia Research Institute (IJC), Badalona, Spain.; ^4^ Tissue Repair and Regeneration Laboratory (TR2Lab), Institut de Recerca i Innovació en Ciències de la Vida i de la Salut a la Catalunya Central (IrisCC), Ctra. de Roda, 70, 08500 Vic Catalunya, Spain.; ^5^ Faculty of Health Sciences and Welfare, University of Vic–Central University of Catalonia (UVic-UCC), C/ Miramarges, 6, 08500 Vic Catalunya, Spain.; ^6^ Department of Genetics and the Human Genetics Institute of New Jersey, Rutgers University, 145 Bevier Road, Piscataway, NJ 08854, USA.; ^7^ Unitat de Citologia i Histologia, Departament de Biologia Cel·lular, Fisiologia i Immunologia, Universitat Autònoma de Barcelona, Bellaterra, Spain.; ^8^ Proteomics Unit, Josep Carreras Leukaemia Research Institute (IJC), Badalona, Spain.; ^9^ The Walter and Eliza Hall Institute of Medical Research, Parkville, Victoria 3050, Australia.; ^10^ Department of Medical Biology, University of Melbourne, Parkville, Victoria 3050, Australia.; ^11^ Department of Structural and Molecular Biology, Instituto de Biología Molecular de Barcelona (IBMB), Consejo Superior de Investigaciones Científicas (CSIC), Barcelona 08028, Spain.; ^12^ Department of Cardiac Development and Remodeling, Max Planck Institute for Heart and Lung Research, D-61231 Bad Nauheim, Germany.; ^13^ Institute for Genetics, Cologne Excellence Cluster on Cellular Stress Responses in Aging-Associated Diseases (CECAD), D-50931 Köln, Germany.; ^14^ Department of Science, Borough of Manhattan Community College (BMCC), The City University of New York (CUNY), New York, NY 10007, USA.; ^15^ Bioinformatics and Medical Statistics Group, Faculty of Science, Technology and Engineering, University of Vic–Central University of Catalonia, 08500 Vic Catalunya, Spain.

## Abstract

Mitosis entry is tightly regulated by a complex network of mechanisms involving epigenetic modifications, signaling pathways, transcriptional control, and structural changes. Although substantial progress has been made in understanding these processes individually, the mechanisms integrating chromatin dynamics with mitotic regulators are still not fully understood. Here, we identify a functional antagonism between the deacetylase SIRT2 and the acetyltransferase MOF in the G_2_-M transition and mitotic progression. This interplay, which involves MOF deacetylation by SIRT2, regulates key histone marks, including H4K16ac (histone 4 lysine-16 acetylation) deacetylation and H4K20me1 (histone 4 lysine-20 monomethylation) deposition, condensin II loading, and the stability of the key mitotic regulator PLK1, and contributes to the FOXM1-mediated transcriptional control of mitosis. Our findings reveal a previously unrecognized layer of regulation in G_2_-M progression with possible impact in cancer, highlighting the intricate cross-talk between chromatin dynamics and mitotic control and providing important insights into chromosomal stability.

## Teaser

The interplay between SIRT2 and MOF regulates G_2_-M transition.

## INTRODUCTION

The G_2_-M transition is a key step in the regulation of cell cycle, genome stability, and cell physiology that involves several interconnected regulatory mechanisms to ensure a proper and efficient transition to mitosis. During the transition from G_2_ to early M, two major chromatin-related events are essential: reorganization of chromosome structure leading to hypercompaction fully reached in metaphasic chromosomes and the specific and tightly controlled regulation of gene expression (*1*–*4*). Although these mechanisms have been extensively studied separately, the underlying regulatory mechanisms that integrate these events are not fully understood.

Chromatin compaction is a complex process involving a general loss of permissive histone marks, mainly acetylation marks and especially histone 4 lysine-16 acetylation (H4K16ac), associated with a concomitant deposition of histone 4 lysine-20 monomethylation (H4K20me1), and general replacement of cohesins by the condensin II complex (*5*). H4K16ac is a hallmark of open chromatin structure involved in gene expression, DNA repair, cancer, and aging (*6*–*9*). When present, this epigenetic mark is associated with active transcription, and it inhibits the folding of the chromatin fiber (*10*). In contrast, H4K20me1, catalyzed by PR-Set7 and primarily removed by PHF8, plays a crucial role in metaphase chromosome compaction, mitotic progression, DNA replication, and gene expression (*11*–*25*). H4K20me1 is recognized specifically by NCAP-D3 and NCAP-G2, two components of the condensin II complex, favoring condensin II loading to chromatin and chromatin compaction (*12*).

The main negative regulator of H4K16ac in G_2_-M is SIRT2, a member of the sirtuin family of nicotinamide adenine dinucleotide (oxidized form) (NAD^+^)–dependent deacetylases, involved in stress response, metabolic homeostasis, and genome stability (*26*–*32*). All available data suggest that SIRT2 has two complementary functions in mitotic entry. On one hand, SIRT2 plays a basal role by deacetylating H4K16ac and facilitating H4K20me1 deposition through several mechanisms, including regulation and recruitment of PR-Set7 (*27*). On the other hand, SIRT2 appears to function as a checkpoint regulator under stress, inducing a block in G_2_-M before progression into prophase (*32*). Consistently, although cells lacking SIRT2 show a faster progression through mitosis, they accumulate DNA damage and develop higher rates of polyploidies and a wide range of chromosomal aberrations.

In contrast, the functional involvement of the main mammalian H4K16 acetyltransferase, MOF (also known as MYST1 or KAT8), during G_2_-M is poorly understood. MOF is an essential gene in mice and has been involved in gene expression, DNA damage response, genome instability, and cell cycle progression (*33*–*35*). Although some evidence suggested that MOF deficiency results in a block of G_2_-M progression (*36*), the role of MOF in this process has not been fully addressed.

The other major event in mitotic entry is gene expression regulation. Whereas chromatin condensation is taking place, global transcription is generally repressed (*2*, *37*–*40*). However, there is a fine-tuned regulation of certain genes crucial for mitotic entry and proper progression. The conserved serine-threonine kinase PLK1 and the transcription factor FOXM1 are central players in this process. PLK1 plays an essential role in mitosis entry as well as in spindle formation, chromosome segregation, and cytokinesis, through the phosphorylation of a wide range of targets, including the phosphatase Cdc25c, cyclin B1, condensin II subunits, or transcription machinery proteins (as FOXM1), among others (*41*–*46*). PLK1 directly activates FOXM1 through phosphorylation at specific sites, allowing FOXM1 to bind to DNA and activate the expression of genes required for mitotic progression such as cyclin B1, aurora kinases, Cdc25, or PLK1 itself (*41*). Dysregulation of the PLK1-FOXM1 axis can lead to several problems in cell division, including mitotic errors, with major consequences in chromosomal instability and cell viability (*47*–*50*).

Here, we have identified and characterized an antagonistic interplay between SIRT2 and MOF in the regulation of mitotic entry, involving cross-talk among key regulatory events, including epigenetic, signaling, and structural mechanisms. Our evidence suggests a role for the SIRT2/MOF axis within the regulatory network governing the G_2_-M transition.

## RESULTS

### SIRT2 and MOF have antagonistic roles in the regulation of G_2_-M transition

**Fig. 1.**
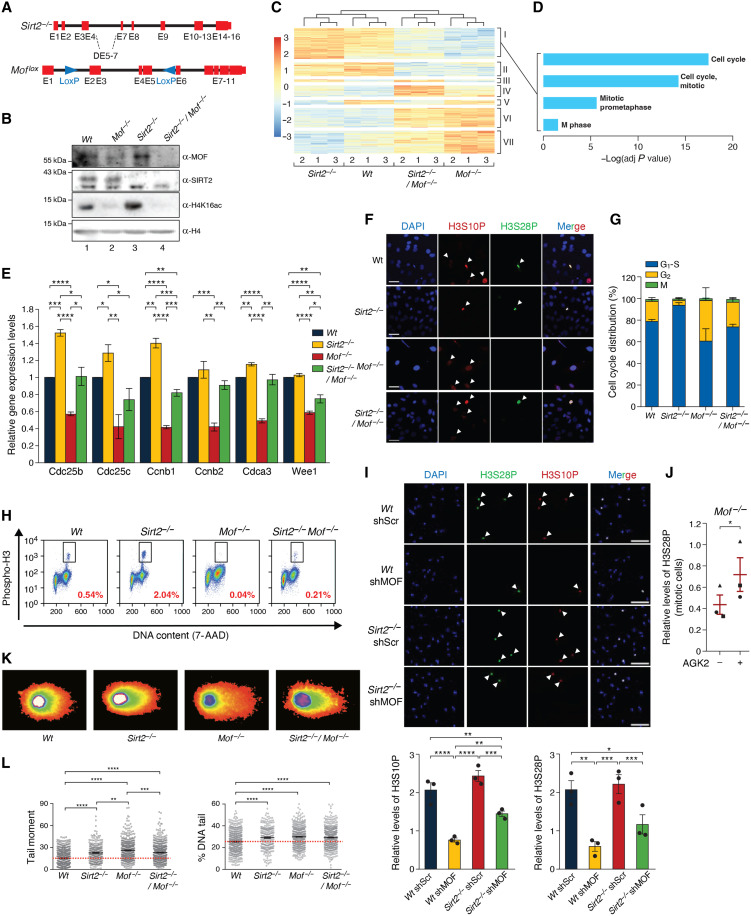
SIRT2 and MOF play antagonistic roles in G_2_-M control. (**A**) Schematic of the generation of *Sirt2^−/−^* and *Mof*^^loxP/loxP^^ mice. For *Sirt2^−/−^* mice, integration of a neomycin cassette led to deletion of exons 5 and 6 and part of exon 7, whereas in *Mof*^^loxP/loxP^^ mice, two *loxP* sites were introduced between exons 1 and 2 and 5 and 6, resulting in deletion of exons 2 to 5 after Cre recombination. (**B**) MOF, SIRT2, and H4K16ac levels measured by Western blot in *Wt*, *Mof^−/−^*, *Sirt2^−/−^*, and *Sirt2^−/−^/Mof^−/−^* primary MEFs (histone H4, loading control). (**C** and **D**) Unsupervised clustering of differentially expressed genes (FDR < 0.05) and GO enrichment for cluster I. (**E**) RT-qPCR analysis of G_2_-M genes in *Wt*, *Mof^−/−^*, *Sirt2^−/−^*, and *Sirt2^−/−^/Mof^−/−^* primary MEFs (means ± SEM, *n* = 3 independent experiments, one-way ANOVA with the Tukey multiple comparisons test). (**F**) Representative immunofluorescence images of H3S10P and H3S28P with DAPI counterstaining (scale bars, 50 μm; *n* = 3). (**G**) Quantification of cell cycle distribution of the primary MEFs based on H3S10P and H3S28P staining (means ± SEM, *n* = 3, ≥1000 cells per condition). (**H**) Representative FACS plots of mitotic cells identified by H3 phosphorylation with DNA content monitored by 7-AAD. (**I**) Immunofluorescence analysis of H3S10P and H3S28P in *Wt* and *Sirt2^−/−^* HeLa cells with MOF down-regulated by shRNA [scale bars, 100 μm; means ± SEM, *n* = 3, ≥50 cells per condition, one-way ANOVA with uncorrected Fisher’s least significant difference (LSD) test]. (**J**) FACS quantification of the relative number of mitotic cells in *Mof^−/−^*-immortalized MEFs treated or not with the SIRT2 inhibitor AGK2 (means ± SEM, *n* = 3, paired one-tailed *t* test). (**K** and **L**) DNA damage in the primary MEFs measured by neutral comet assay shown as representative images and quantification of the tail moment and percentage of DNA in tail (means ± SEM, *n* = 3, ≥50 cells per condition, one-way ANOVA with the Tukey multiple comparisons test). *P < 0.05; **P < 0.01; ***P < 0.001; ****P < 0.0001.

Aiming to understand the interplay between both H4K16ac-associated enzymes, we generated a double SIRT2/MOF knockout (KO) mice, by crossing *Sirt2*^−/−^ mice with inducible *Mof*^−/−^ mice [Mof(lox/lox)/CAGG-Cre^ERT^(T/+)] (Fig. 1A and fig. S1, A and B) (*27*, *33*, *51*). As expected, loss of MOF upon treatment with 4-hydroxytamoxifen (4-OHT), which induces MOF knockdown through activation of the estrogen receptor system, resulted in a drastic H4K16 hypoacetylation whereas SIRT2-deficient mouse embryonic fibroblasts (MEFs) harbored a mild increase in global H4K16ac levels, reflecting the specific regulation of this mark by SIRT2 during late G_2_ to early mitosis. *Sirt2*^−/−^/*Mof*^−/−^ showed global H4K16 hypoacetylation comparable to *Mof*^−/−^ cells (Fig. 1B and fig. S1C), confirming the dominant effect of MOF in the maintenance of H4K16ac levels. We next studied the impact of SIRT2/MOF antagonism in gene expression through RNA sequencing (RNA-seq) analysis of *Wt*, *Sirt2*^−/−^, *Mof*^−/−^, and *Sirt2*^−/−^/*Mof*^−/−^ primary MEFs. All four cell populations clustered separately in principal components analysis (PCA), including *Sirt2*^−/−^/*Mof*^−/−^, indicating a clear impact of the interplay between both enzymes in transcription (fig. S1D). In the gene expression analysis, we were able to identify seven different clusters (Fig. 1C), the larger of which (cluster I) contained 4096 genes and was enriched in genes associated with cell cycle and mitotic entry regulation (Fig. 1D and fig. S1E). As predicted, MOF deficiency resulted in down-regulation of the genes of the cluster I, whereas SIRT2 loss had the opposite effect. *Sirt2*^−/−^/*Mof*^ −/−^ cells showed a compensatory effect between both factors (Fig. 1C), supporting a functional antagonism between both proteins in the regulation of these genes. In agreement with a specific involvement of both factors in the regulation of G_2_-M, SIRT2 and MOF had opposite effects on the expression of key regulatory factors involved in G_2_-M transition but not G_1_-S (Fig. 1E and fig. S1F).

We next confirmed the impact of the SIRT2/MOF antagonism on G_2_-M progression. To this end, we monitored the levels of two mitosis-related histone marks, phosphorylation of H3S28 (H3S28P), a mitotic marker, and phosphorylation of H3S10 (H3S10P), a marker of mid-late G_2_ and mitosis, in *Wt*, *Sirt2*^−/−^, *Mof*^ −/−^, and *Sirt2*^−/−^/*Mof*^ −/−^ MEFs. As predicted, whereas we detected mitotic staining with both marks in *Wt* and *Sirt2*^−/−^ MEFs, no H3S28P-positive cells were observed in *Mof*^ −/−^ MEFs. Notably, this mitotic block was not observed when MOF KO was induced in *Sirt2*^−/−^ MEFs, strongly supporting a functional antagonism of both factors in G_2_-M control (Fig. 1, F and G). We also performed a similar analysis with fluorescence-activated cell sorting (FACS) using mitotic phospho-H3 marker and 7-amino-actinomycin D (7-AAD). The results again confirmed that SIRT2 deficiency partially prevents the G_2_-M mitotic block induced by induction of MOF knockdown (Fig. 1H). We further confirmed these results by short hairpin RNA (shRNA)–mediated down-regulation of MOF in *Wt* or *Sirt2^−/−^* HeLa cells generated by CRISPR-Cas9 technology (Fig. 1I and fig. S1, G and H). As in the case of primary MEFs, although MOF down-regulation in *Wt* cells resulted in a drastic decrease in the number of mitotic H3S10P and H3S28P-positive cells, this effect was partially reverted when MOF was down-regulated in *Sirt2*^−/−^ cells (Fig. 1I).

We next tested whether chemical inhibition of SIRT2 using the specific inhibitor AGK2 could restore mitotic progression in MOF-deficient cells. The efficiency of SIRT2 inhibition under these conditions (2 μM) was confirmed by hyperacetylation of α-tubulin, a well-established SIRT2 substrate unrelated to H4K16ac regulation (fig. S1, I and J) (*52*). To this end, AGK2 was administered either both before and during MOF KO induction with 4-OHT (ALLTIME-SIRT2i) or only after MOF KO induction (POST-SIRT2i) (Fig. 1J and fig. S1, K and L). In agreement with a role for SIRT2 activity in MOF-dependent regulation of G_2_-M, the inhibition of SIRT2 activity before and during induction of MOF knockdown (ALLTIME-SIRT2i) was able to prevent the mitotic block induced by MOF deficiency (monitored by H3S28P) (Fig. 1J and fig. S1K). AGK2 treatment after MOF depletion did not have any effect, suggesting that the block induced by MOF depletion is irreversible (fig. S1L). Comet assays showed that, although the loss of either factor led to the accumulation of DNA damage, the combined loss of both did not rescue this phenotype, suggesting that the impact of the SIRT2/MOF interplay is not related to DNA repair per se but rather to the signaling of the G_2_-M checkpoint (Fig. 1, K and L). Together, our evidence strongly supports a functional antagonism between both factors in the control of mitotic entry, which, in turn, has a direct impact on genome stability.

### SIRT2 and MOF interplay regulates H4K20me1 deposition

**Fig. 2.**
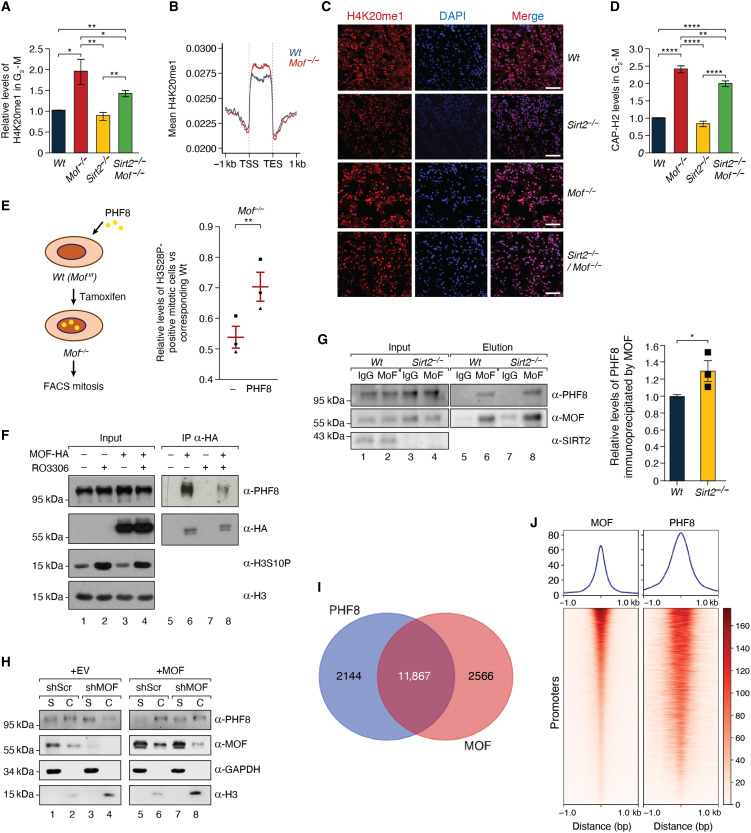
The interplay between SIRT2 and MOF regulates H4K20me1 deposition and condensin loading in G_2_-M. (**A**) Quantification of H4K20me1 levels measured by FACS in G_2_-M *Wt*, *Mof^−/−^*, *Sirt2^−/−^*, and *Sirt2^−/−^/Mof^−/−^* primary MEFs identified by PI staining (means ± SEM, *n* = 3, one-way ANOVA with the Tukey multiple comparisons test). (**B**) H4K20me1 ChIP-seq signal across annotated genes in Wt and *Mof^−/−^* primary MEFs. TSS, transcription start site; TES, transcription end site. (**C**) Immunohistochemical detection of H4K20me1 in kidney cryosections from indicated genotypes with DAPI nuclear counterstaining (scale bars, 50 μm). (**D**) CAP-H2 levels in G_2_-M *Wt*, *Mof^−/−^*, *Sirt2^−/−^*, and *Sirt2^−/−^/Mof^−/−^* primary MEFs measured by FACS (means ± SEM, *n* = 3, one-way ANOVA with the Tukey multiple comparisons test). (**E**) Experimental overview and FACS quantification of mitotic cells (H3S28P-positive) in *Mof^−/−^*-immortalized MEFs infected or not with PHF8 (means ± SEM, *n* = 3 shown independently, paired two-tailed *t* test). (**F**) HA immunoprecipitation of endogenous PHF8 from HEK293F cells transfected with MOF-HA or empty vector and treated or not with RO3306. (**G**) MOF immunoprecipitation in *Wt* and *Sirt2^−/−^* HeLa cells with interacting PHF8 relative levels quantified by Western blot (means ± SEM, *n* = 3 shown independently, unpaired one-tailed *t* test). (**H**) Fractionation analysis of PHF8 distribution between soluble and chromatin-bound fractions upon MOF shRNA-mediated depletion or expression. Controls for the soluble fraction (GAPDH) and chromatin fraction (histone H3) are also shown. (**I**) Venn diagram of overlapping MOF and PHF8 ChIP-seq peaks in HepG2 cells. (**J**) ChIP-seq analysis showing the genomic occupancy of MOF (left) and PHF8 (right) around the TSS in HepG2 cells. Heatmaps show the normalized number of reads across a 1-kb genomic interval relative to the TSS. Composite plots above each heatmap quantify the mean signal. *P < 0.05; **P < 0.01; ****P < 0.0001.

Considering the proposed antagonism between H4K16ac and H4K20me1, and the described role of SIRT2 in deposition of H4K20me1 during G_2_-M, in part through deacetylation of H4K16ac (*27*), we hypothesized that the antagonism between SIRT2 and MOF may be important in the control of H4K20me1 in mitotic entry. Immunofluorescence experiments in asynchronized cells showed that *Mof^ −/−^* MEFs harbored decreased levels of H4K16ac (fig. S2A). As previously observed by Western blot, the levels of H4K16ac in *Mof^ −/−^*/*Sirt2^−/−^* MEFs were not significantly altered compared to *Mof^−/−^* cells (fig. S2A). Notably, we observed the opposite effect on H4K20me1 levels in these cells with a stronger compensatory effect between both factors. The significant hypermethylation of H4K20me1 observed in MOF-deficient MEFs suggested a crucial and undescribed role of MOF in the regulation of this modification (fig. S2A). We detected a significant antagonism in the regulation of H4K20me1 in G_2_-M between SIRT2 and MOF (Fig. 2A) in MEFs, suggesting that this antagonistic interplay was important during mitosis. In agreement with this antagonism, chromatin immunoprecipitation sequencing (ChIP-seq) experiments showed that the loss of MOF generally resulted in H4K20me1 hypermethylation in gene bodies (Fig. 2B and fig. S2B). Congruently, shRNA-driven down-regulation of MOF increased H4K20me1 levels and MOF overexpression rescued the levels of the mark (fig. S2C). Further supporting this antagonism between both enzymes in vivo, immunofluorescence analysis of kidney tissues derived from *Wt*, *Mof^−/−^*, *Sirt2^−/−^*, and *Mof^−/−^*/*Sirt2^−/−^* mice showed that, although the levels of H4K20me1 in *Sirt2^−/−^* tissues were significantly lower than *Wt* and *Mof^−/−^* animals harbored significantly higher levels of H4K20me1 (Fig. 2C and fig. S2D). Loss of both proteins in vivo compensated for each other’s effects as the levels of H4K20me1 observed in *Sirt2^−/−^*/*Mof^−/−^* animals were comparable to those in *Wt* tissues (Fig. 2C and fig. S2D). In early mitosis, H4K20me1 was previously found to also regulate the deposition of condensins, key structural factors involved in metaphase chromosome compaction, through the specific binding of condensin II subunits NCAP-D3 and NCAP-G2 to this histone mark (*12*). In agreement with the opposed interplay between MOF and H4K20me1, MOF absence resulted in premature accumulation of the levels of condensin II (Fig. 2D). SIRT2 and MOF had opposing effects on condensin II accumulation in G_2_-M, although MOF appeared to have a stronger impact than SIRT2. Consistent with our observations for H4K20me1, a compensatory effect was detected in *Mof^−/−^*/*Sirt2^−/−^*, supporting an antagonistic relationship between the two factors.

Given the importance of H4K20me1 in mitotic entry and our observations indicating that its regulation is a key function of MOF during G_2_-M, we hypothesized that its dysregulation may contribute to the G_2_-M cell cycle arrest observed upon MOF deficiency. To test that, we analyzed whether the H4K20me1 hypermethylation observed in *Mof^−/−^* MEFs could be rescued by overexpression of PHF8, the main H4K20me1 demethylase, that was previously involved in cell cycle control (*12*, *53*). Notably, we observed a significant increase in the proportion of mitotic cells in *Mof^−/−^* cells upon PHF8 overexpression, supporting our hypothesis (Fig. 2E and fig. S2, E- to G). Considering that PHF8 was identified as an interactor of NSL, a MOF-containing protein complex, through HCF1 (*12*), we next studied whether MOF and PHF8 factors interact. Thus, we confirmed the interaction between MOF and PHF8 in asynchronous cells (Fig. 2F). This interaction was reduced in cells arrested in early mitosis with the CDK1 inhibitor RO3306 (Fig. 2F and fig. S2H), which suggested that the functional interplay between MOF and PHF8 is no longer valid once H4K20me1 is established in mitosis. Although no direct interaction was observed between SIRT2 and PHF8 (fig. S2I), the interaction between MOF and PHF8 was boosted in SIRT2-deficient cells, suggesting that SIRT2 regulates this interaction through MOF (Fig. 2G). Moreover, although MOF down-regulation resulted in a specific decrease in PHF8 chromatin enrichment, its reintroduction restored PHF8 levels, supporting a direct control of MOF on PHF8 localization (Fig. 2H and fig. S2K). Comparison of MOF and PHF8 genomic distribution showed that both shared more than 82% of target genes (82.23% MOF; 84.7% PHF8), most of them in promoters and genic regions and related to cell cycle, supporting a close functional interplay between both enzymes (Fig. 2, I and J, and fig. S2, K and L).

### MOF deacetylation by SIRT2 inhibits it catalytic activity

**Fig. 3.**
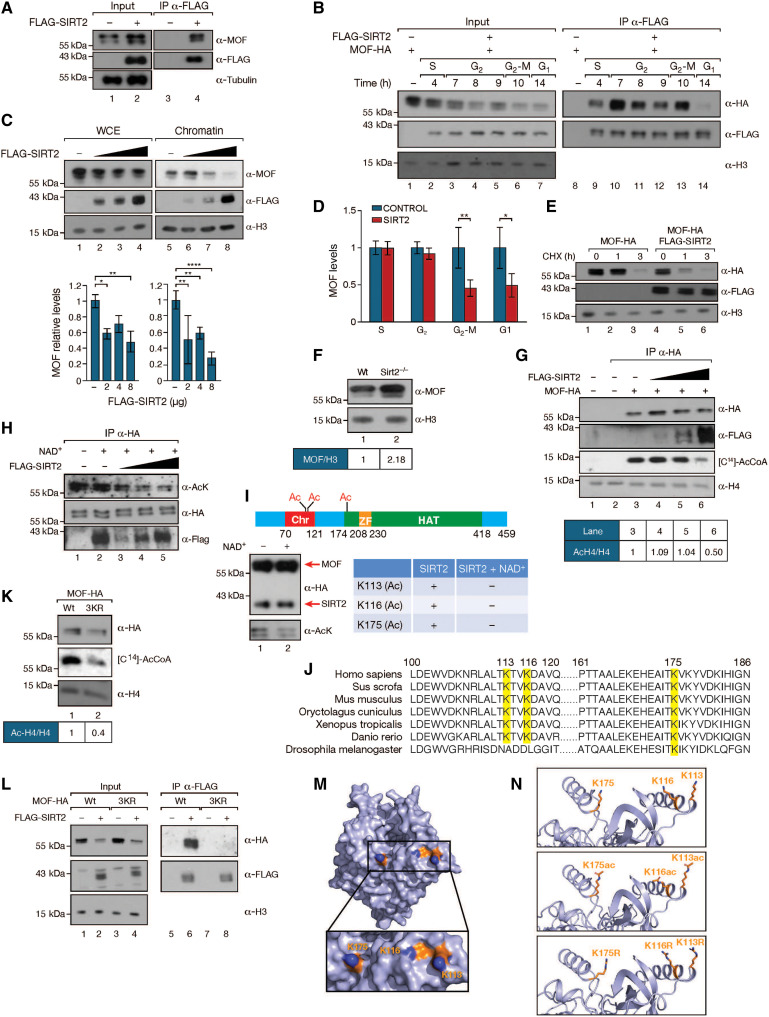
SIRT2 binds to and deacetylates MOF impairing its catalytic activity. (**A** and **B**) FLAG immunoprecipitation in HeLa cells expressing FLAG-SIRT2 of endogenous (A) or overexpressed MOF-HA in cells synchronized with double thymidine block (B). h, hours. (**C**) Endogenous MOF levels analyzed in whole-cell extracts (WCE) and chromatin from NIH/3T3 cells −/+ increasing amounts of FLAG-SIRT2. Loading control: histone H3. Quantifications is shown as means ± SEM; *n* = 4 independent experiments, one-way ANOVA with Dunnett’s post hoc test. (**D**) Overexpressed MOF levels assessed by Western blot in HeLa cells −/+ SIRT2 at different cell cycle stages (means ± SD, *n* = 3 independent experiments, unpaired two-tailed *t* test). (**E**) MOF-HA and SIRT2-HA protein levels expressed and analyzed by Western blot in HeLa cells treated with CHX (100 μg/ml) for 0, 1, or 3 hours. Loading control: histone H3. (**F**) Endogenous MOF levels in *Wt* and *Sirt2^−/−^* primary MEFs analyzed by Western blot and quantified by densitometry normalized to histone H3. (**G**) In vitro acetylation assays performed using purified MOF-HA from HeLa cells transfected −/+ increasing amounts of FLAG-SIRT2, core histones, and [^14^C]acetyl-CoA. Autoradiography and Coomassie staining of histone H4 are shown, with densitometric quantification normalized to H4 and expressed relative to MOF alone. (**H**) In vitro deacetylation assays performed using purified MOF as a substrate and increasing amounts of purified SIRT2 in the presence of NAD^+^, with acetylation monitored by anti–acetyl-lysine(α-AcK) Western blot. (**I**) Schematic representation of MOF showing identified acetylated lysines by MS (top) and validation by in vitro deacetylation assays as in (H) (bottom). Chr, chromodomain; ZF, zinc fingers; HAT, acetyltransferase domain. (**J**) Sequence alignment of conserved MOF lysines K113, K116, and K175. (**K**) In vitro acetylation assays (as in G) comparing MOF-WT and MOF-3KR, with autoradiography and densitometric quantification of [^14^C]-H4/H4 normalized with MOF levels. (**L**) FLAG immunoprecipitation of FLAG-SIRT2 coexpressed with MOF-WT-HA or MOF-3KR-HA in HeLa cells. (**M** and **N**) Structural modeling of whole WT hMOF with K113, K116, and K175 labeled (M) or the region around these residues unacetylated, acetylated, or mutated to arginine (N). *P < 0.05; **P < 0.01; ****P < 0.0001.

Supporting a direct link between both factors, immunoprecipitation experiments in HeLa cells confirmed the interaction between SIRT2 and MOF (Fig. 3A). In vitro SIRT2 pull-down experiments demonstrated that the interaction between both factors was direct (fig. S3A) and involved the C-terminal HAT domain of MOF (fig. S3, B and C). This interaction peaked to early G_2_ and to G_2_-M in thymidine block synchronized cells (Fig. 3B and fig. S3, D and E). This not only confirmed a direct link between both factors in G_2_-M but also suggested their interplay in the postreplicative stage, probably related to their proposed role in DNA damage response signaling (*27*, *29*, *54*, *55*). Supporting this antagonism, we also observed that SIRT2 overexpression induced a global decrease in MOF levels, especially in chromatin fractions (Fig. 3C). Immunoprecipitation analyses of synchronized cells indicated that this effect occurs exclusively during G_2_-M and G_1_ (Fig. 3D and fig. S3, F and G). These observations correlated with a decrease in MOF protein stability, as observed in experiments incubating with the translation inhibitor cycloheximide (CHX) upon overexpression of FLAG-SIRT2 (Fig. 3E and fig. S3H). No changes were observed in MOF mRNA levels (fig. S3I). Consistently, SIRT2-deficient MEFs harbored higher MOF protein levels than *Wt* MEFs (Fig. 3F). The enzymatic activity of MOF purified from HeLa cells expressing increasing amounts of SIRT2 was inversely proportional to SIRT2 expression levels (Fig. 3G). Moreover, the incubation of MOF with SIRT2 in the presence of NAD^+^ resulted in a decrease in acetyllysine levels on MOF, indicating that the SIRT2-dependent reduction in MOF activity was due to deacetylation (Fig. 3H and fig. S3J). Mass spectrometry (MS) analysis of MOF incubated with SIRT2, in the presence or absence of NAD^+^, identified three MOF residues that were deacetylated by SIRT2 (Fig. 3I, and fig. S3, K and L) Two of them (Lys^113^ (K113) and Lys^116^ (K116)) were located in the chromodomain of MOF, whereas the third one (Lys^175^ (K175)) was located at the beginning of the HAT catalytic domain (Fig. 3I). K113 and K116 are conserved residues across all vertebrates, whereas K175 is conserved in all MOF orthologs, including that of *Drosophila* (Fig. 3J). Mutation of all three lysine residues to arginine (MOF 3KR), which mimics nonacetylated lysine, resulted in a significant decrease in MOF enzymatic activity, suggesting that SIRT2 regulates MOF activity through deacetylation (Fig. 3K). Mutation of these residues abrogated SIRT2 binding to MOF (Fig. 3L), indicating an important role of these three lysines in SIRT2 binding to MOF. Structural analysis of a three-dimensional model of human MOF, based on available structures, revealed that residues K113, K116, and K175 are located in closely positioned, solvent-exposed regions of the protein, with their side chains oriented outward from the protein core (Fig. 3M). Comparison of the wild-type (WT) unacetylated and acetylated K113/K116/K175 models, as well as the 3KR mutant model, showed no detectable conformational changes at either the global or local level, with no substantial alterations in overall fold or local geometry, indicating preservation of structural integrity (Fig. 3N). These observations suggest that K113, K116, and K175 are unlikely to play a structural role in maintaining MOF folding stability. Instead, their surface exposure and tolerance to substitution are consistent with a functional role in modulating protein-protein interactions. Together, these results suggest that SIRT2 exerts a dynamic control on MOF function through deacetylation, and this is a key event in the regulation of mitotic progression.

### Mutation of the *Mof* residues deacetylated by SIRT2 recapitulates the impact of MOF deficiency on G_2_-M regulation

**Fig. 4.**
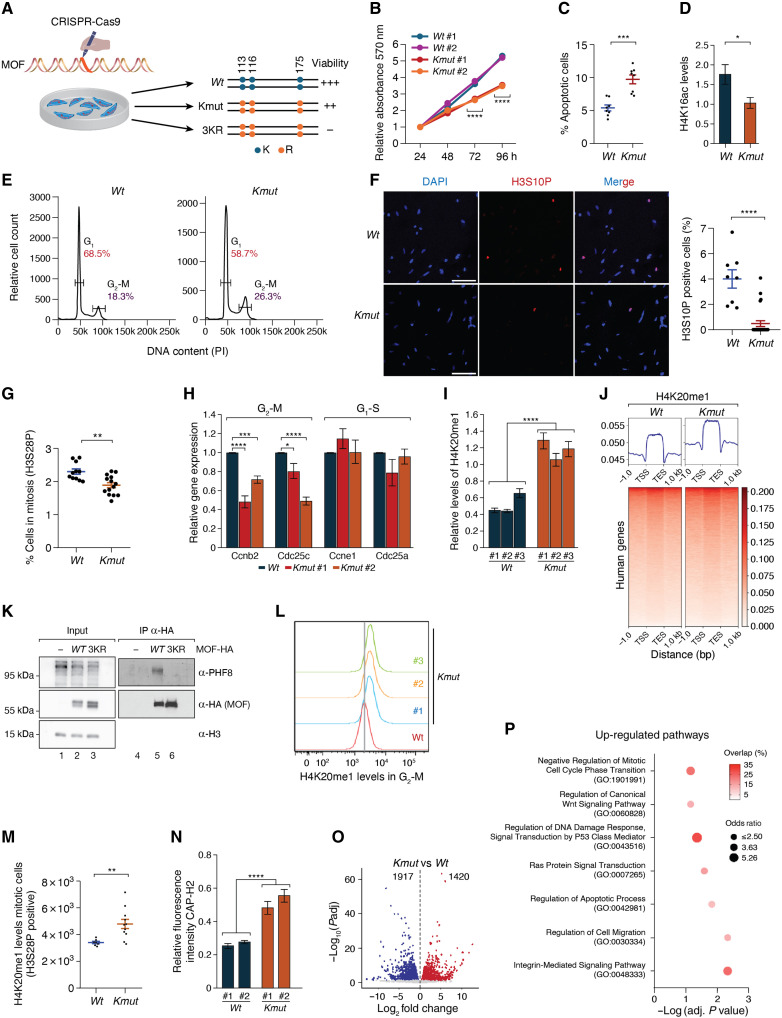
Mutation of *Mof* residues targeted by SIRT2 alters G_2_-M epigenetic progression, gene expression, and cell cycle progression. (**A**) CRISPR-Cas9 gene targeting of *Mof* gene generated three clones: *W*t (K113/K116/K175), *Kmut* (homozygous R113/R116/heterozygous K175/R175), and 3KR (homozygous R113/R116/R175); viability is indicated. (**B**) MTT assay of *Wt* and *Kmut* cells (means ± SEM, *n* = 3, two clones per condition, two-way ANOVA with the Sidak multiple comparisons test). (**C**) Apoptotic annexin V/PI assay in *Wt* and *Kmut* cells. Apoptotic cells included annexin V+/PI− and annexin V+/PI+ cells (means ± SEM, *n* = 3, three clones per condition, unpaired two-tailed *t* test). (**D**) Relative levels of H4K16ac in *Wt* and *Kmut* (means ± SEM, *n* = 3, three clones per condition, unpaired two-tailed *t* test). (**E**) PI cell cycle analysis in *Wt* and *Kmut*. (**F**) H3S10P levels in *Wt* and *Kmut* by immunofluorescence including representative images (scale bars, 100 μm) and H3S10P-positive quantification (means ± SEM, *n* = 3, >100 cells per condition, unpaired two-tailed *t* test). (**G**) Relative levels of mitotic (H3S28P-positive) *Wt* and *Kmut* cells (FACS) (means ± SEM, *n* = 4, >3 clones per condition, unpaired two-tailed *t* test). (**H**) RT-qPCR of G_2_-M (*Ccnb2* and *Cdc25c*) and G_1_-S (*Ccne1* and *Cdc25a*) genes in *Wt* and *Kmut* (clones #1and #2) (means ± SEM, *n* = 4, one-way ANOVA with the Tukey multiple comparisons test). (**I**) H4K20me1 levels by immunofluorescence in *Wt* and *Kmut* (clones #1 to #3) (means ± SEM, *n* = 3, >25 cells per condition, one-way ANOVA with the Tukey multiple comparisons test). (**J**) H4K20me1 signal intensity at genes bodies in *Wt* and *Kmut*. Heatmaps show normalized reads across a 1-kb genomic interval relative to the TSS and TES. Mean signal shown above each heatmap. (**K**) MOF-WT-HA or MOF-3KR-HA immunoprecipitation of PHF8 from HEK293F. (**L**) Representative FACS plots of H4K20me1 levels in *Wt* and *Kmut* (clones #1 to #3) cells. (**M**) H4K20me1 levels by FACs in *Wt* and *Kmut* mitotic (H3S28P-positive) cells (means ± SEM, *n* = 3, >3 clones per condition, unpaired two-tailed *t* test). (**N**) CAP-H2 levels by immunofluorescence in *Wt* and *Kmut* HeLa cells (means ± SEM, *n* = 3, >25 cells per condition, one-way ANOVA with the Tukey multiple comparisons test). (**O**) RNA-seq volcano plot of up-regulated (1420; blue) and down-regulated (1917; red) genes between *Wt* and *Kmut* cells. (**P**) GO analyses for selected biological processes of up-regulated genes in *Kmut* respect *Wt*. Dot size indicates the ratio of genes per category. *P < 0.05; **P < 0.01; ***P < 0.001; ****P < 0.0001.

To further understand the functional significance of MOF deacetylation by SIRT2, we introduced an endogenous 3KR mutation of *Mof* in HeLa cells through CRISPR-Cas9 technology. In these cells, we generated lysine-to-arginine (K→R) mutations in both *Mof* alleles, either Lys^113^→Arg (K113R)/ Lys^116^→Arg (K116R) or Lys^175^→Arg (K175R), but we were unable to obtain all three mutations together, suggesting that simultaneous mutation of all three residues induces cell lethality. Instead, we were able to generate HeLa cells harboring mutations K113R and K116R in both copies of *Mof*, but K175R in only one of the copies (K113^RR^/K116^RR^/K175^KR^ or *Kmut*) (Fig. 4A and fig. S4A). *Kmut* cells were viable, although they showed decreased viability and increased apoptosis compared to parental *Wt* cells (Fig. 4, B and C). On the other hand, single mutants K113^RR^/K116^RR^ and K175^RR^ showed an intermediate viability between *Kmut* and *Wt* (fig. S4B).

As expected, *Kmut* cells harbored a significant 44% decrease in H4K16ac levels (Fig. 4, D and fig. S4C) and showed an accumulation in the G_2_-M phase of the cell cycle (Fig. 4E). Analysis of the mitotic markers H3S10P and H3S28P in these cells revealed a significant reduction in the mitotic population compared to *Wt* cells (Fig. 4, F and G, respectively), suggesting that these mutations in MOF induced a delay in the progression of these cells through G_2_-M transition before entering mitosis. Single mutants K113^RR^/K116^RR^ and K175^RR^ did not show this effect on cell cycle, indicating that all three residues are required for the role of MOF in mitotic regulation (fig. S4D). In agreement with this, the expression of key regulators of G_2_-M transition cyclin B2 (*Ccnb2*) and *Cdc25c*, but not of G_1_-S regulators cyclin E1 (*Ccne1*) and *Cdc25a*, was specifically down-regulated in *Kmut* cells compared to *Wt* cells (Fig. 4H). Notably, we also observed increased levels of rereplication in *Kmut* cells, further supporting a misregulated state of G_2_-M progression (fig. S4, E and F).

Confirming a direct functional relationship between MOF/SIRT2 interplay and H4K20me1, *Kmut* cells showed a robust global H4K20me1 hypermethylation compared with *Wt* cells in all clones tested (Fig. 4I and fig. S4G). ChIP-seq analysis in *Wt* and *Kmut* cells revealed that the observed increase in H4K20me1 in *Kmut* was associated with H4K20me1 accumulation over gene bodies (Fig. 4J). The genomic distribution of this mark also revealed a relative increase in the number of peaks on intergenic regions (fig. S4H). The mutation of these three lysine residues in *Mof* abrogated the interaction with PHF8 (Fig. 4K), highlighting the important role of the MOF/SIRT2 antagonism in the regulation of H4K20me1.

The global H4K20me1 hypermethylation observed in Kmut cells did not account for the observed changes in gene expression as more than 60% of genes displaying altered H4K20me1 levels were unaffected at the transcriptional level (fig. S4I). These observations suggest that H4K20me1 regulated by the MOF/PHF8 axis is more likely to play a structural role.

Supporting a specific impact in G_2_-M, FACS analysis revealed a significant increase in H4K20me1 levels in G_2_-M *Kmut* cells compared to *Wt* cells across all clones tested (Fig. 4L), whereas no significant changes were observed in individual clones (fig. S4J). An increase in H4K20me1 was also observed in mitotic H3S28P-positive *Kmut* cells, further supporting our observations (Fig. 4M and fig. S4K). Consistently, *Kmut* clones also showed an abnormal global accumulation of condensin II (Fig. 4N and fig. S4G).

RNA-seq analysis of *Kmut* cells compared to *Wt* parental cells identified 1420 genes up-regulated and 1917 genes down-regulated (Fig. 4O and fig. S4L). Gene Ontology (GO) analysis of the expression profile of *Kmut* cells was consistent with the induction of G_2_-M restriction and checkpoint activation as we detected an up-regulation of negative regulators of mitosis and key signaling pathways related to DNA damage response, apoptosis, and cell cycle progression, such as *p53*, *Ras*, or *Wnt* (Fig. 4P). In contrast the down-regulated genes in *Kmut* cells were restricted to metabolism, further supporting an additional role of the MOF/SIRT2 interplay in metabolic regulation (fig. S4M).

### Deacetylation of MOF in Lys^113^, Lys^116^ and Lys^175^ impairs its interaction with key regulators

**Fig. 5.**
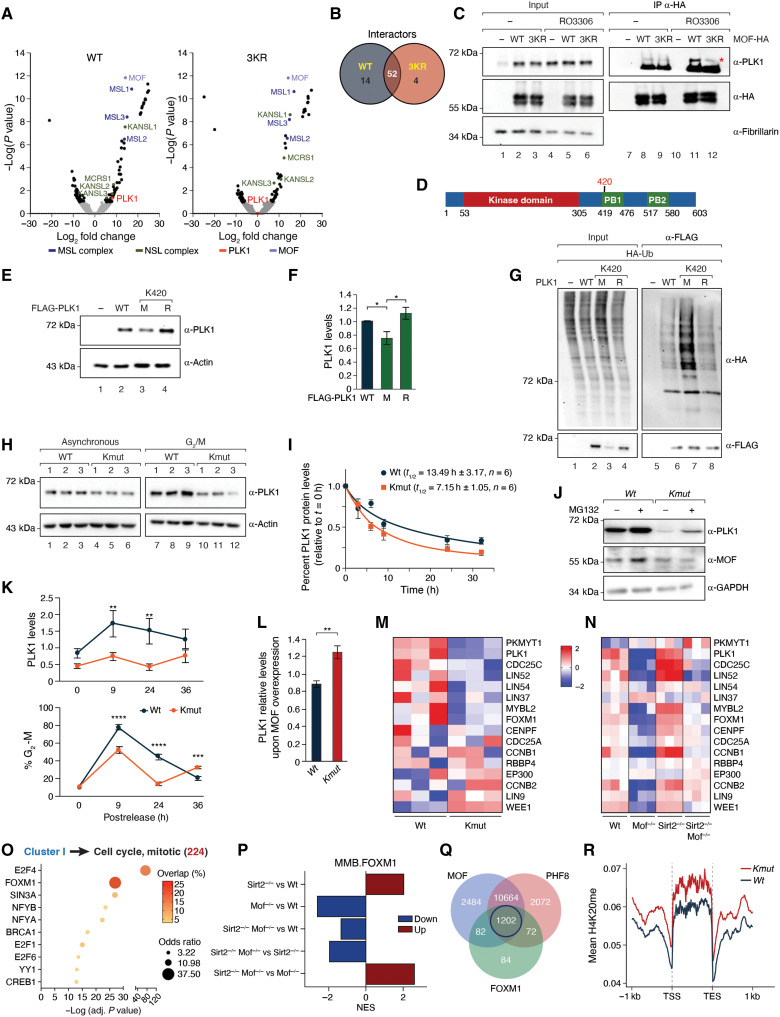
MOF interacts with PLK1 in a SIRT2-dependent manner and regulates its stability. (**A**) Volcano plots of MOF-WT and MOF-3KR interactors. The MS (dark blue) and NSL (green) complexes, PLK1 (red), and MOF (light blue) are indicated. (**B**) Number of common and specific interactors of MOF WT and 3KR. (**C**) MOF-WT-HA or MOF-3KR-HA coimmunoprecipitation of PLK1 in HEK293F cells treated or untreated with RO3306. Asterisk denotes PLK1. (**D**) Diagram of PLK1 indicating the K420 residue identified in G_2_-M–synchronized *Kmut* cells. PB, Polo-box domain. (**E** and **F**) PLK1 WT, K420M, and K420R levels in G_2_-M–synchronized HeLa cells. Representative immunoblot (E) and quantification (F) are shown (means ± SEM, *n* = 4, one-way ANOVA with the Newman-Keuls multiple comparisons test). (**G**) Ubiquitination of purified FLAG-PLK1 K420 mutants in HeLa cells expressing HA-ubiquitin (Ub). (**H**) PLK1 protein levels in asynchronous and G_2_-M in *Wt* and *Kmut* (three clones) cells. (**I**) Relative PLK1 protein levels in *Wt* and *Kmut* cells after incubation of CHX (100 μg/ml) at indicated times. The PLK1-to-actin ratio was normalized relative to time 0 (means ± SEM, *n* = 3, two clones per condition). (**J**) Representative PLK1/MOF Western blots in *Wt* and *Kmut* cells, after 5 μM MG132 treatment for 6 hour. (**K**) PLK1 levels and %G_2_-M population in *Wt* and *Kmut* cells synchronized with double thymidine block (means ± SEM, *n* = 3, two clones per condition, two-way ANOVA with Bonferroni’s multiple comparisons test). (**L**) PLK1 levels in *Wt* and *Kmut* cells −/+ MOF overexpression (means ± SEM, *n* = 3, three clones per condition, unpaired two-tailed *t* test). (**M** and **N**) RNA-seq heatmap of gene expression in the “Polo-like kinase–mediated events” Reactome category in *Wt* and *Kmut* cells (M) and indicated primary MEFs (N). (**O**) Transcription factor enrichment analysis for the cluster I from RNA-seq (Fig. 1C) using Enrichr (ENCODE and ChEA Consensus TFs from ChIP-X). The top 10 hits are displayed. (**P**) FOXM1 Gene Set Enrichment Analysis (GSEA) of RNA-seq gene expression data from the primary MEFs. NES, normalized enrichment score. (**Q**) Venn diagram showing the gene overlap between MOF, PHF8, and FOXM1 ChIP-seq in HepG2 cells. (**R**) H4K20me1 ChIP-seq signal in genes co-occupied by MOF, PHF8, and FOXM1 in *Wt* and *Kmut* cells. *P < 0.05; **P < 0.01; ***P < 0.001; ****P < 0.0001.

Aiming to understand the impact of SIRT2-dependent deacetylation of MOF, we next performed a comparative interactome analysis between the MOF WT and the MOF 3KR mutant. For that purpose, we expressed the WT and 3KR mutant in human embryonic kidney (HEK) 293F cells, purified them by affinity chromatography, and performed a label-free MS analysis of MOF interactors (fig. S5A). The results identified 66 and 56 statistically significant interactors of MOF WT and MOF 3KR proteins, respectively, 52 of which were common for both. Meanwhile, 14 and 4 proteins were only detected in WT or 3KR purification (Fig. 5, A and B). Among the 52 common interactors, members of the MOF-containing MSL and NSL complexes, cell cycle and G_2_-M checkpoint regulators, p53-related factors, and DNA repair factors were identified (Fig. 5B and fig. S5B). Among the 14 exclusive interactors of MOF WT, we identified PLK1, a master regulator of G_2_-M progression (*56*) (fig. S5C). Immunoprecipitation experiments of cells treated or not with the CDK1 inhibitor RO3306, which arrests cells in G_2_-M, confirmed the preferential interaction between MOF and PLK1 during G_2_-M, which was abrogated in the case of the 3KR mutant (Fig. 5C and fig. S5, D and E).

To understand the role of MOF on PLK1, we purified FLAG-PLK1 from G_2_-M arrested *Wt* and *Kmut* cells after RO3306 treatment and analyzed differences in the pattern of posttranslational modifications of PLK1 between both cell lines. We observed no significant differences in acetylation. However, we detected monomethylation of Lys^420^ (K420) in PLK1 in two of three *Kmut* cells replicates but not in *Wt* cells (Fig. 5D and fig. S5, F and G). This PLK1 residue is highly conserved from yeast to human and is located next to the consensus cleavage site for caspase 3 DYSD (fig. S5F) (*57*). Aiming to understand the impact of this PTM in PLK1 in mitotic entry, we expressed PLK1 WT and two PLK1 K420 mutants HeLa cells: PLK1 Lys^420^→Arg (K420R), which mimics unmodified lysine, and PLK1 Lys^420^→Met (K420M), which mimics methylated lysine. Analysis of these proteins in G_2_-M–enriched cells showed that K420M resulted in a significant decrease in PLK1 levels, whereas K420R have the opposite effect on PLK1 levels (Fig. 5, E and F). PLK1 K420M mutation was associated with a robust increase in PLK1 polyubiquitination (Fig. 5G and fig. S5H), suggesting that MOF binding to PLK1 prevents K420 methylation and protects PLK1 from ubiquitination and subsequent degradation.

These observations suggest that MOF regulates PLK1 stability during G_2_-M. In agreement with these findings, we observed that *Kmut* cells harbor significantly lower levels of PLK1 in G_2_-M compared to *Wt* parental cells, whereas no significant differences were observed in asynchronous cells (Fig. 5H and fig. S5I). Considering the detected interaction between both factors, we hypothesized that the interaction with MOF regulates PLK1 protein stability. To test this possibility, we performed CHX timing experiments on endogenous PLK1 in HeLa *Wt* and *Kmut* cells. The results showed a significant 47% decrease in the half-life of PLK1 in *Kmut* cells compared to *Wt* cells (Fig. 5I and fig. S5J). Moreover, treatment with the proteasome inhibitor MG132 led to increased levels of both PLK1 and MOF in *Wt* cells, whereas in *Kmut* cells, MG132 did not alter MOF levels and had only a minor effect on PLK1 (Fig. 5J). These findings not only suggest that proteasome-mediated degradation of PLK1 is somehow impaired in *Kmut* cells but also further reinforced that MOF is a regulator of PLK1 stability. Supporting these findings, synchronized HeLa *Wt* and *Kmut* cells showed that *Kmut* cells not only harbored lower endogenous PLK1 levels but also failed to up-regulate PLK1 in G_2_-M (9 hours), which correlated with a decrease in the number of cells entering mitosis (Fig. 5K and fig. S5, K and L). We further confirmed that the impact of MOF on PLK1 was direct as overexpression of MOF in *Kmut* cells restored PLK1 levels (Fig. 5L and fig. S5M). Together, our evidence suggests that MOF regulates PLK1 during G_2_-M.

### The functional interplay between MOF and PLK1 regulates FOXM1-dependent transcription during G_2_-M

To further understand the functional relationship between MOF and PLK1, we analyzed one of the signatures associated with PLK1, Polo-like kinase–mediated events, in RNA-seq experiments from MEFs and HeLa cells. This showed a down-regulation of PLK1-associated genes, including PLK1 itself, in *Mof*^−/−^ and *Kmut* cells (Fig. 5, M and N). In agreement with these results, we also studied the transcription factor enrichment in cluster I of the MEF RNA-seq and FOXM1 was identified as the major hit (Fig. 5O). FOXM1, a member of the DREAM complex, activates the G_2_-M transcriptional program after PLK1 phosphorylation (*41*).

Consistently, we detected a global repression of FOXM1-associated genes (*58*) in *Mof^−/−^* cells compared to *Wt*, whereas SIRT2 deficiency had the opposite effect (Fig. 5P). Notably, loss of MOF in *Sirt2^−/−^* cells had a significantly milder impact on these FOXM1 genes, further supporting that the MOF/SIRT2 antagonism regulates G_2_-M through PLK1/FOXM1 signaling (Fig. 5P and fig. S5N). Analysis of previously reported ChIP-seq experiments revealed that MOF and FOXM1 share 1284 common genes, the vast majority of which are also occupied by PHF8 (Fig. 5Q). GO analysis of these common genes indicated that most are associated with cell cycle and mitosis regulation (fig. S5O), further supporting a functional interplay between these factors in regulation of mitotic entry. In agreement with our previous evidence, genes co-occupied by MOF, PHF8, and FOXM1 displayed increased levels of H4K20me1 in *Kmut* and *Mof^-/-^* primary MEFs compared to their respective *Wt* controls (Fig. 5R and fig. S5P, respectively).

### The SIRT2/MOF antagonism has an impact on cancer

**Fig. 6.**
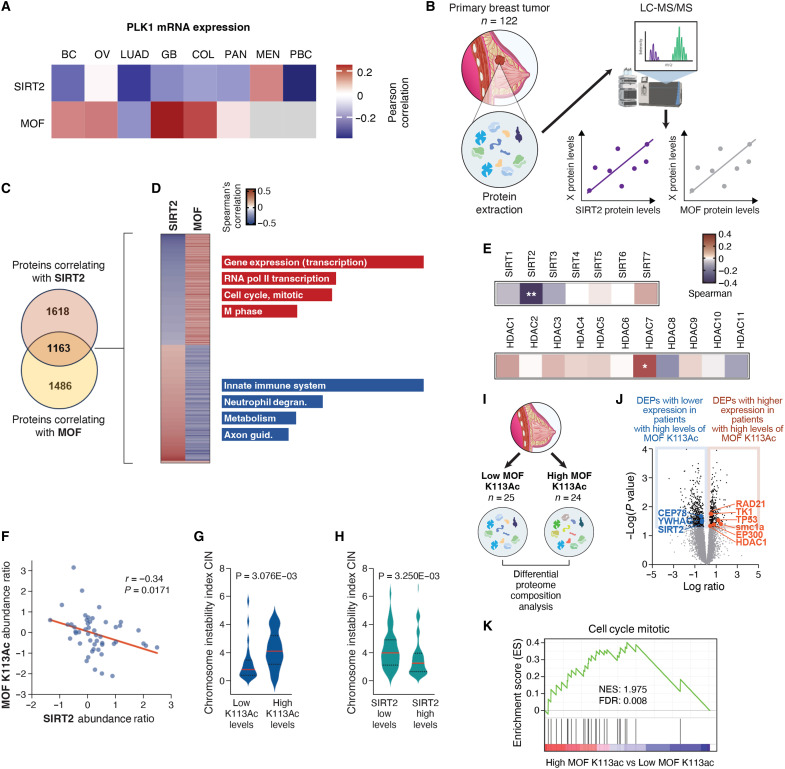
The antagonism between SIRT2/MOF affects genome instability and cancer. (**A**) Heatmap of the correlation between *PLK1* mRNA levels and MOF and SIRT2 protein levels in breast cancer (BC), ovarian serous cystadenocarcinoma (OV), lung adenocarcinoma (LUAD), glioblastoma (GB), colon cancer (COL), pancreatic ductal adenocarcinoma (PAN), meningioma (MEN), and pediatric brain cancer (PBC). (**B**) Schematic diagram of the approach. The proteomic profile of 122 primary breast tumors was used to perform correlation analysis between the expression levels of proteins identified in the tumors and SIRT2 or MOF protein levels. LC-MS/MS, liquid chromatography–tandem mass spectrometry. (**C**) Venn diagram representing proteins that significantly correlate with both SIRT2 and MOF protein levels. (**D**) Expression level heatmap and GO analysis of the 1163 proteins that significantly correlate with both SIRT2 and MOF protein levels. Degran., degranulation; guid., guidance. (**E**) Correlation analysis of MOF-K113 acetylation (MOF K113ac) levels and histone deacetylases protein levels in primary breast tumors for which matched acetylproteomics and proteomics data were available (*n* = 49). (**F**) Correlation plot of MOF K113ac levels and SIRT2 protein levels in matched tumors. (**G** and **H**) CIN in primary breast tumors with low MOF K113ac levels (*n* = 25) or high MOF K113ac levels (*n* = 24) (G) and CIN in primary breast tumors with low SIRT2 protein levels (*n* = 44) or high SIRT2 proteins levels (*n* = 74) (H). Violin plots including the median (red bar) and the interquartile range (dashed lines). Statistical differences were assessed applying the Wilcoxon test. (**I**) Differential proteomic analysis was conducted on 49 primary breast tumors stratified by low (*n* = 25) or high (*n* = 24) MOF K113ac levels. (**J**) Volcano plot of differentially expressed proteins (DEPs) in tumors with high MOF K113ac levels. In these patients, 356 and 549 proteins were identified as significantly up-regulated or down-regulated, respectively. (**K**) “Cell cycle mitotic” enrichment plot based on differentially expressed proteins between breast cancer tumors with high or low MOF K113ac levels.

Considering our results and the well-established roles of MOF and SIRT2 in genome stability and cancer, we next analyzed the in vivo relevance of their functional interplay using previously reported proteomic and expression datasets across a wide range of cancer types. Specifically, we examined the correlation between SIRT2 and MOF protein levels and *PLK1* mRNA expression. Although some variability was observed, this analysis revealed a generally opposing pattern, with SIRT2 displaying a negative correlation and MOF displaying a positive correlation with *PLK1* expression (Fig. 6A). Among the cancer types in which this inverse relationship reached statistical significance, we focused on breast cancer. This choice was motivated by its strong association with genome instability and the availability of comprehensive acetylome and proteome datasets from patients with breast cancer.

Analysis of a comprehensive publicly available Clinical Proteomic Tumor Analysis Consortium (CPTAC) proteogenomic dataset of patients with breast cancer (*59*) showed a clear inverse correlation between SIRT2 and MOF in these cancer samples. We identified 1163 proteins whose expression correlated either directly or inversely with both SIRT2 and MOF levels (Fig. 6, B and C). In most of these proteins (>98.7%), their expression correlated inversely with SIRT2 compared to MOF, supporting a functional relevance of their antagonism in cancer (Fig. 6D). Whereas MOF positively correlated with proteins involved in RNA polymerase II transcription, cell cycle, and mitotic regulation, SIRT2 showed an inverse correlation with these same protein networks. In contrast, SIRT2 positively correlated with genes related to the immune system and metabolism, among others (Fig. 6D).

We next analyzed the involvement of acetylation/deacetylation of the three MOF residues targeted by SIRT2. Of them, only K113ac was detected in the acetylproteome data of this breast cancer cohort. In these studies, the levels of SIRT2, but not any other HDAC or sirtuin, showed a significant inverse correlation with MOF K113ac (Fig. 6, E and F, and fig. S6, A and B). When we divided the cohort into patients with low versus high levels of K113ac, we observed a notable significant direct correlation between this modification and chromosome instability index (CIN) levels in these tumors (Fig. 6G). A similar analysis with SIRT2 protein levels resulted in a significant opposite correlation between SIRT2 and CIN (Fig. 6H). We compared the proteome profile between the patient cohort samples with high and low MOF K113ac levels, which showed 905 differentially expressed proteins between both groups, with 356 proteins up-regulated and 549 proteins down-regulated in high samples versus low samples (Fig. 6, I and J). Normalized enrichment score (NES) analysis identified a cell cycle mitosis hallmark as one of the significantly altered pathways between them (Fig. 6K and fig. S6C). In this hallmark, among the proteins up-regulated in high MOF K113ac, we identified p53, thymidine kinase 1 (TK1), cohesin complex subunits (RAD21 and SMC1A), HDAC1, or the HAT p300, among others. In contrast, SIRT2, the chaperone YWHAG, and the centrosome regulator CEP78 were some of the down-regulated proteins (fig. S6C). Together, our results strongly support a crucial role of the MOF/SIRT2 regulatory axis in the regulation of G_2_-M progression with important implications for genome stability and cancer research.

## DISCUSSION

**Fig. 7.**
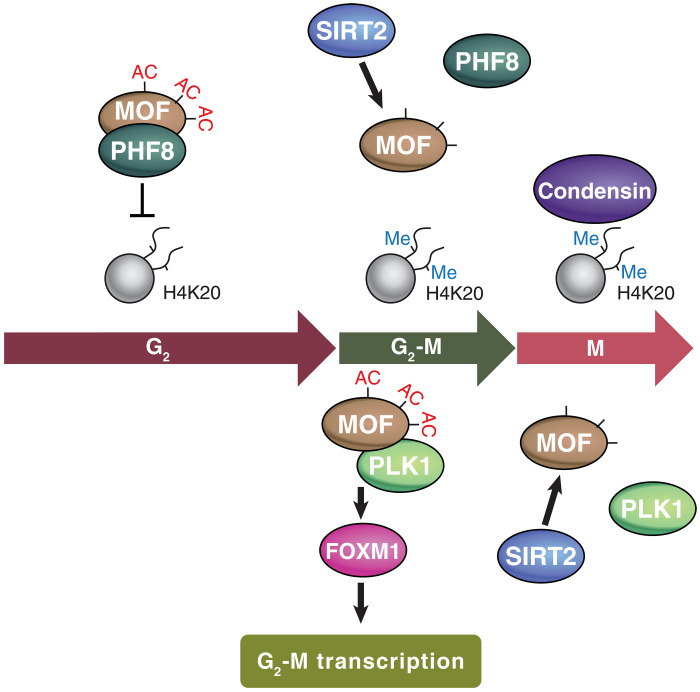
Proposed model illustrating the interplay between SIRT2 and MOF in the control of the G2-M transition based on our findings. The model summarizes how SIRT2-mediated deacetylation of MOF modulates H4K20me1 deposition, condensin loading, PLK1 stability, and chromosomal architecture during G_2_-M progression, thereby affecting genome stability and tumorigenesis.

Our study identifies a functional antagonism between SIRT2 and MOF in the regulation of G_2_-M transition and mitotic entry. Overall, our evidence suggests that the SIRT2-MOF antagonism plays an important role in coordinating mitotic entry through the integration of epigenetics, chromatin structure, and transcription. On the basis of our observations, we propose that, during G_2_-M, SIRT2 negatively regulates MOF activity and protein stability, leading to global H4K16ac hypoacetylation and H4K20me1 hypermethylation, decreased cell viability, and accumulation in G_2_-M (Fig. 7). The role of MOF in mitotic entry has been poorly characterized. Our findings not only provide a previously unidentified perspective on the function of MOF in cell cycle regulation but also reinforce, and notably expand, our previous reports describing the role of SIRT2 in the deacetylation of H4K16ac and the deposition of H4K20me1 during G_2_-M. Together, these results reveal an unknown level of interplay between chromatin marks and their regulatory enzymes, offering an integrated view of how SIRT2 and MOF coordinate chromatin dynamics to ensure proper cell cycle progression at the G_2_-M transition. In this context, SIRT2 targets three specific residues in MOF: two in the chromodomain and one in the catalytic HAT domain. Cells harboring knock-in mutations of these residues to arginine (*Kmut*) not only exhibit reduced MOF activity but also display an altered MOF interactome profile, including key proteins involved in mitotic regulation, such as the H4K20me1 demethylase PHF8 and the kinase PLK1. *Kmut* cells recapitulate many of the effects induced by SIRT2, ranging from G_2_-M progression to the establishment of the mitotic epigenetic profile, suggesting that this is one of the main signaling pathways through which SIRT2 regulates G_2_-M. Reflecting the relevance of this uncharacterized mechanism, analysis of publicly available data from patients with breast cancer further supports an antagonism between these factors, as well as a significant inverse correlation between SIRT2 and MOF K113ac, which may have a direct impact on genome instability and cancer. Supporting their link to PLK1 signaling regulation, SIRT2 and MOF appear to correlate in opposite directions with PLK1 mRNA expression across several cancer types. Nevertheless, although important, this association requires further validation to determine whether deacetylation of these three residues in vivo has a direct role in cancer. The intermediate phenotype observed in the *Sirt2^−/−^ Mof^−/−^* double mutant highlights the complex functional relationship between these factors during the G_2_-M transition. Although this phenotype may suggest partial compensation, our data indicate that the interplay between MOF and SIRT2 is important for mitotic progression, whereas both factors also exert additional functions beyond their defined roles in chromatin regulation and mitotic entry, as illustrated, for example, by the lack of rescue in DNA damage responses (Fig. 1, K and L). Interpretation of the double-loss phenotype is further complicated by the largely irreversible impact of MOF depletion in primary cells, which limits the ability of SIRT2 inhibition or PHF8 expression to fully compensate for MOF loss and restore all MOF-dependent functions.

One of the most intriguing findings of this work is the major impact of the SIRT2/MOF interplay on H4K20me1. These findings align with the reported antagonism between H4K16ac and H4K20me1 in chromatin structure (*5*, *16*), particularly during mitosis (*15*), and with our group’s previous work demonstrating SIRT2-dependent induction of H4K20me1 deposition in G_2_-M through direct interaction and deacetylation of the main H4K20me1 HMT, PR-Set7 (*27*). However, our observations suggest a more complex regulation of this key epigenetic mark. We show that MOF not only regulates H4K20me1 through the deposition of the antagonistic mark H4K16ac but also through the recruitment of the H4K20me1 demethylase PHF8. The global genomic colocalization of MOF and PHF8 supports a close functional relationship between these factors in gene expression and genome structure. Recent reports have also identified HR23A and B as H4K20me1-3 demethylases (*11*). Although we cannot discount the possibility that MOF also regulates H4K20me1 through these demethylases, the involvement of PHF8 and MOF in the FOXM1-dependent DREAM complex suggests that all these factors work together to regulate entry into mitosis. It is also relevant to note that our findings indicate that SIRT2 regulates the interplay between MOF and PHF8 as the 3KR mutant of MOF abrogates its interaction with PHF8, and loss of SIRT2 results in increased binding between these factors. However, whether SIRT2 may regulate PHF8 through additional mechanisms, or whether PHF8 could compensate for the effects of SIRT2 deficiency during G_2_-M progression, remains unknown and should be addressed in future studies. The role of PHF8 in these processes is further supported by a previous study showing that PHF8 loss results in a longer G_2_ phase and defective mitosis (*60*). The massive loading of condensin II during prophase was previously found to require H4K20me1 (*12*), suggesting that the hypermethylation of H4K20me1 induced by MOF deficiency leads to abnormal and premature accumulation of the condensin complex, providing an explanation for the mitotic block observed in MOF KO cells. Notably, we observed that the G_2_-M block in MOF KO cells is irreversible (fig. S1L), reinforcing the drastic impact of MOF function on chromosome dynamics before mitotic entry. The finding that *Kmut* cells show early deposition of H4K20me1 and condensin complexes further suggests that SIRT2 regulates the role of MOF in mitotic epigenetics and chromosomal structure not only through deacetylation of H4K16ac but also through modification of the three residues.

It is also important to note that our evidence suggests that the interplay between SIRT2 and MOF in the regulation of H4K20me1 is not globally linked to gene expression but rather to a well-defined structural role in chromatin compaction and mitotic entry (*12*). In this context, H4K20me1 has been proposed to exert dual functions in gene regulation, being associated with both gene activation and repression (*13*, *23*–*24*). On the basis of our findings, we hypothesize that the major mechanism by which MOF regulates the expression of FOXM1/DREAM complex target genes is largely dependent on H4K16ac, consistent with the reduced H4K16ac levels observed in Kmut MOF cells. In contrast, we propose that PHF8 plays two distinct roles in this regulatory framework: a general role, together with MOF, in preventing H4K20me1 accumulation and condensin loading before the G_2_-M transition and a second, more dynamic role involving localized H4K20me1 demethylation at specific loci during cell cycle progression. Future studies will be required to define the precise contribution of PHF8 in this context and to elucidate its role in regulating the DREAM complex transcriptional program throughout the cell cycle. Another major finding in our work is the direct role of MOF in regulating PLK1 during mitosis. Our results suggest that PLK1 signaling is directly regulated by the antagonistic interplay between MOF and SIRT2. The fact that *Kmut* cells also show a similar PLK1 expression signaling profile than MOF-deficient cells indicates that the interaction between MOF and PLK1 is a key event in the regulation of PLK1 signaling. Whereas the DREAM complex regulates the expression of PLK1 and the transcriptional program during G_2_-M, PLK1 has also been shown to regulate the transcriptional activity of the complex through the phosphorylation of some of its components, especially FOXM1 (*41*). Thus, the MOF-PLK1 interaction may also play an important role, not only in PLK1 stability and activity during mitotic entry but also in the control of G_2_-M expression through the DREAM complex. Future studies should determine the relevance of this interplay. At the molecular level, we have identified a protective role for MOF in PLK1 protein stability, at least in part through the regulation of methylation at Lys^420^. This modification was previously identified, along with five other residues, as a target of lysine monomethylation by G9a (*61*). In that report, only one of these residues, Lys^209^, was characterized, showing that it inhibits PLK1 activation in G_2_-M, thereby delaying mitotic progression. Similarly, our evidence suggests that Lys^420^ also promotes PLK1 inactivation by regulating PLK1 stability. This residue is located in the Polo-box 1 domain of PLK1, which has previously been reported to be important for localization in mitotic chromosomes and centrosomes (*62*), interaction with a wide range of factors, and especially for substrate recognition (*63*). We could not identify any MOF-dependent acetylation in PLK1 (fig. S5C) through proteomic analysis, which suggests that MOF blocks Lys^420^ methylation through its binding to PLK1. Lys^420^ is located in one of the most conserved regions among PLK1 orthologs, between the first and second β sheet. Considering the relevance of this lysine in the domain, the addition of a methylation group should alter markedly the packing of these β sheets, suggesting an inhibitory effect on PLK1 activity. Thus, we hypothesize that a defective binding of MOF to PLK1 allows the access of other factors, among which is probably G9a. Because mutation of Lys^420^ to Met (K420M) induced a decreased protein stability and higher levels of polyubiquitination, it suggests that this modification is a prerequisite for the binding of a ubiquitin ligase mediating this polyubiquitination and subsequent proteasome-induced degradation. Previous reports have reported on the interaction between PLK1 and APC, the major regulator of cell cycle, which suggests that it may be involved. Future studies should define the specific details of this mechanism, including the implication of methylation and the identity of the E3 ubiquitin ligase.

Together, our findings place the SIRT2/MOF axis at the intersection of multiple regulatory layers—including epigenetic, signaling, and structural—highlighting its crucial contribution to the regulation of a timely and accurate G_2_-M transition and genome stability (Fig. 7, K and L).

## MATERIALS AND METHODS

### Mouse models

The *Sirt2*^*−/−*^ C57BL/6 mice used in these experiments were generated in Tong’s lab using the original embryonic stem (ES) cells generated by D. Alt’s lab, as previously described (*27*). The transgenic conditional Mof KO mice were obtained from A.K.V.’s lab. They generated the *Mof*-deficient CRE C57BL/6 strain by breeding a *Mof(loxP/+)* male (*33*) into a CAGG-Cre-ERT(T/+) transgenic female as previously described (*51*, *64*). To generate the *Sirt2*^*−/−*^ Mof-deficient CRE C57BL/6 strain, *Sirt2*^*−/−*^ mice were crossed to *Mof(loxP/loxP-cre)* mice. We considered that we got a stable strain after five generations of cross-breeding.

Genomic DNA for mice genotyping was extracted from ear punches, and standard polymerase chain reaction (PCR) was performed using the following oligonucleotides: SIRT2 A, 5′-GACTGGAAGTGATCAAAGCTC-3′; SIRT2 B, 5′-CAGGGTCTCACGAGTCTCATG-3′; SIRT2 C, 5′-CAAATCTGGCCAGAACTTATG-3′; MOF A, 5′-TATCTGCCTTTCTCTGTCAATGGG-3′; MOF B, 5′-AGGTGAGCCAGGTTAGGACTTGG-3′; MOF C, 5′-TGGCACACACCTTTAGATCCACC-3′; CAGGs, 5′-CTCTAGAGCCTCTGCTAACC-3′; and Cre, 5′-CCTGGCGATCCCTGAACATGTCC-3′. The sizes of the PCR products of WT and SIRT2 KO alleles are 538 and 700 base pairs (bp), respectively. For MOF PCR, we obtained a 422-bp fragment for WT, whereas the insertion of two loxP sites increased the size of the PCR fragment to 509 bp for the floxed allele (flox). The same primers amplified a 277-bp fragment for the null allele. The size of the amplified product in the case of cre gene is ~300 bp.

Mice were treated with tamoxifen (TM) (Sigma-Aldrich, T5648) dissolved in corn oil (Thermo Fisher Scientific, 10616051) at a concentration of 10 mg/ml. For analysis of adult mice, corn oil alone or 6 mg of TM per 40 g of body weight was injected intraperitoneally for 7 consecutive days. Two weeks after the last injection (or, in some cases, after premature animal death), various organs from the mice were fixed and subjected to histochemical staining.

All mice were bred at the Comparative Medicine and Bioimage Centre of Catalonia animal facility of the Germans Trias i Pujol Research Institute. Animal studies were conducted at the Josep Carreras Leukemia Research Institute (IJC) (Spain) according to national/regional authorities (Generalitat de Catalunya, project authorization for animal experimentation no. 10472) and institutional ethics committees (Germans Trias i Pujol Research Institute Ethics Committee, 19-030-AMU).

### Generation of the SIRT2 KO HeLa cell line

A SIRT2 KO HeLa cell line was generated using the CRISPR-Cas9 system. Briefly, specific single guide RNA (sgRNA) sequences were designed using the Zhang laboratory CRISPR design tool (https://crispor.gi.ucsc.edu) (CACCGCTACCTGCGTGTAGCAGCGC and CACCGCGCAGAGTCATCTGTTTGGT) and cloned into a pSpCas9(BB)-2A-GFP (PX458) expression vector (Addgene, 48138) containing a green fluorescent protein (GFP) cassette. HeLa cells transfected with these vectors were sorted by GFP with a FACS Aria II (BD Biosciences) and seeded one cell per well in 96-well plates to obtain isogenic clones. The presence of gene-disrupting indels in edited cell lines was confirmed by Sanger sequencing, and the ablation of protein production was assessed by immunoblotting.

### Generation of the MOF point mutant HeLa cell line

To mutate Lys^113^, Lys^116^ and Lys^175^ to Arg (R) in HeLa cells, we used the Alt-R CRISPR HDR Design Tool of IDT (Integrated DNA Technologies). We designed specific crRNAs (Lys^113^-Lys^116^ crRNA, GGCGCUGACCAAGACAGUGAGUUUUAGAGCUAUGCU; and Lys^175^ crRNA, GAUCACCAAGGUGAAGUAUGGUUUUAGAGCUAUGCU) that hybridizes with a universal tracrRNA (IDT, 1072532) to form a functional sgRNA duplex to activate the Cas9 enzyme (IDT, 1081058) and a specific HDR (homology- directed repair) donor template oligo: one for the Lys^113^ and Lys^116^ (GCGGCTGGACGAGTGGGTAGACAAGAACCGGCTGGCGCTGACCCGTACAGTGCGTGATGCTGTACAGAAGAACTCAGAGAAGTACCTGAGCGAGCTCGCA) and another one for Lys^175^ (TGCTGAGCCGTGCACTGTGCCTCACTCCCACCCTGCCTGCAGATCACCCGTGTGAAGTATGTGGACAAGATCCACATCGGGAACTACGAAATTGATGCC). 

First, we prepared the sgRNA complex annealing crRNA and tracrRNA to a final concentration of 50 μM, and then, we combined sgRNA and Cas9 nuclease to form the ribonucleoprotein (RNP) complex to a final concentration of 4 μM Cas9:5 μM sgRNA. Last, we prepared cells for nucleofection using the Cell Line Nucleofector kit R (Lonza Bioscience VCA-1001) and Program I-013 of the Lonza Nucleofector Transfection 2b Device. The transfection mix contained a cell suspension in nucleofector solution, RNP complex, HDR donor oligo, and Alt-R Cas9 electroporation enhancer (IDT, 1075915) to improve delivery. The next day, we performed limiting dilution cloning and expanded clones until we have enough number of cells to perform genomic DNA isolation to detect mutations by DNA sequencing.

### Cell lines and culture conditions

Primary MEFs were derived from E13.5 mouse embryos, following standard protocols. Briefly, the uterus containing the embryos was extracted from the pregnant female and placed in phosphate-buffered saline (PBS) (Gibco, 10010023) supplemented with antibiotic-antimycotic solution (Sigma-Aldrich, A5955). Each embryonic sac was dissected individually and processed as an independent cell line. The embryonic body, excluding the head and liver, was placed in 1 ml of 0.125% trypsin-EDTA (Gibco, 15400054) and incubated overnight at 4°C. The next day, partially digested embryos were vortexed to dissociate the cells and plated in 150-mm culture dishes. Cells were cultured in Dulbecco’s modified Eagle’s medium (DMEM) (Gibco, 11965092) supplemented with 10% fetal bovine serum (FBS) (Gibco, 10270106), penicillin/streptomycin (P/S) (100 U/ml; BioWest, L0022), 1 mM sodium pyruvate (BioWest, L0642), 1% nonessential amino acids (BioWest, X0557), at 37°C in humidified atmosphere with 5% CO_2_. Primary MEFs were cultured until passage 5 to prevent cell cycle arrest and cellular senescence. Primary MEFs were immortalized as previously described (*65*, *66*). To promote the access of Cre recombinase to the nucleus to initiate recombination of LoxP sites and delete the Mof gene before each experiment, MEFs were cultured in the presence of 1 μM 4-OHT (Sigma-Aldrich, H7904) dissolved in methanol for 72 hours.

HeLa (female), HEK293T (female), and NIH/3T3 (male) cells were maintained in DMEM supplemented with 10% FBS and P/S (100 U/ml) in a humidified incubator under 5% CO_2_ and 37°C.

### Cell culture treatments

For SIRT2 inhibition, cells were treated with 2 μM AGK2 (Sigma-Aldrich, A8231) for 72 hours. For protein degradation assays, media were supplemented with CHX (100 μg/ml; Sigma-Aldrich, 01810). For proteasome inhibition treatment, cells were treated with 5 μM MG132 (Selleck Chemicals, S2619) for 6 hours.

HeLa cells were synchronized at the G_1_-S boundary by using double thymidine block. Briefly, cells were treated with 2 mM thymidine (Sigma-Aldrich, T1895) for 18 hours, then released into fresh medium for 10 hours, and blocked again with 2 mM thymidine for 12 hours. Last, cells were released from the block into fresh medium, and time points were taken at different times. To arrest cells in G_2_-M transition, cells were treated with 10 μM RO3308 (Sigma-Aldrich, SML0569) for 20 hours.

### Transfection

HeLa, HEK293F, and NIH/3T3 cells were transiently transfected with different expression vectors using 3 μl of polyethylenimine (1 mg/ml; Polysciences, 23966) per μg of DNA. The following plasmids were used for transfection: pCMV4-FLAG, pCMV4-FLAG-SIRT2, pCDNA4/T0-HA, pCDNA4/T0 SIRT2-HA, pCDNA4/T0 MOF-HA, pCDNA4/T0-MOFK113R-K116R-K175R-HA, pCDNA4/T0 MOFΔH-HA, pCDNA4/T0 MOFΔC-HA, pCDNA4/T0 MOFHAT-HA, pCDNA4/T0 FLAG-Plk1 (this vector was subcloned from the Addgene plasmid #41160), pCDNA4/T0 FLAG-PLK1-K420M, pCDNA4/T0 FLAG-PLK1-K420R, and pCDNA4/T0-Ub-HA (a gift from H. Piwnica-Worms). The different constructs harboring point mutations were cloned by site-directed mutagenesis and then verified by sequencing.

Gene expression knockdown was carried out using 2.5 × 10^5^ cells that were transfected with the ON-TARGETplus Human SIRT2 (22933) siRNA SMARTpool (Horizon Discovery, L-004826-00-0005), siGENOME Mouse Kat8 siRNA SMARTpool (Horizon Discovery, M-048962-00-0005), or nontargeting control pool (Horizon Discovery, D-001810-10-20) using DharmaFECT 1 transfection reagent (Horizon Discovery, T-2001-02) according to the supplier’s protocol. Briefly, 15 μl of small interfering RNA (siRNA; 20 μM) was incubated with 8.5 μl of DharmaFECT in DMEM for 20 min, and then the transfection mix was added to the cells in a 100-mm dish.

### Lentiviral production and infection

Lentiviruses were produced by transfecting HEK293T cells with indicated vectors using polyethylenimine. The medium was replaced with fresh media at least 6 hours after transfection, and 48 hours later, the viral supernatant was collected and filtered. Last, immortalized MEFs or HeLa cells were infected with viral supernatants for 24 hours three times in a row. The following plasmids were used for infection: pLVX-IRES-ZsGreen1 HA, pLVX-IRES-ZsGreen1 HA PHF8 (this vector was subcloned from the vector pEV833 PHF8 from M. Martínez-Balbás), pLKO.1-Scrambled, and pLKO.1-shRNA targeting MOF designed using the sequences from the TRC shRNA library [pLKO-shRNA MOF exon 1 (5′-GCAAGATCACTCGCAACCAAA-3′), pLKO-shRNA MOF exon 3 (5′-CGAAATTGATGCCTGGTATTT-3′), shRNA MOF 3-UTR A (CTCCCAGCCTGTAAATATGT), shRNA MOF 3-UTR B (GTCGGACCTGAGCCTCCTGG), and shRNA MOF 3-UTR C (CTGGTGGCCCTGGACTTTGG)].

### Recombinant protein purification

The *Escherichia coli* BL21 strain transformed with vectors pET30b-rSIRT2 or pGEX-MOF-GST (a gift from A. Akhtar) was cultivated overnight at 37°C in LB and inoculated to 1 liter of fresh LB for amplification until reaching 0.6 OD (optical density). To induce protein expression, isopropyl-β-d-thiogalactopyranoside (IPTG) (1 mM, final concentration) was added to the culture for 3 hours at 37°C with agitation. Harvested cells were resuspended in NETN buffer [20 mM Tris (pH 7.8), 100 mM NaCl, 1 mM EDTA, 0.5% NP-40, and 0.2% sarcosyl] and lysed by sonication.

For recombinant SIRT2, after centrifugation at 15,000 rpm at 4°C for 15 min, the cleared lysates were loaded onto a Ni-NTA agarose (Qiagen, 30210) column and incubated at 4°C for 30 min to allow protein binding. After several washes with NETN buffer and BC500 [20 mM tris-HCl (pH 8.0), 500 mM NaCl, 10% glycerol, 1 mM EDTA, 1 mM dithiothreitol (DTT), 0.1% NP-40, and 0.1 mM phenylmethylsulfonyl fluoride (PMSF)], rSIRT2 was slowly eluted with 100 mM imidazole and subsequently dialyzed in BC100 [20 mM tris-HCl (pH 8.0), 100 mM NaCl, 10% glycerol, 1 mM EDTA, 1 mM DTT, 0.05% NP-40, and 0.1 mM PMSF].

For recombinant MOF (rMOF), after centrifugation at 15,000 rpm at 4°C for 15 min, the cleared supernatant was loaded onto a Glutathione Sepharose (Sigma-Aldrich, GE17-0756-01) column and incubated for 30 min. After several washes with NETN buffer and TST buffer [50 mM tris-HCl (pH 7.8), 150 mM NaCl, and 0.1% Triton], rMOF was eluted using 20 mM l-glutathione reduced.

### Immunoprecipitations

Whole-cell extracts were obtained using radioimmunoprecipitation assay (RIPA) buffer [50 mM tris-HCl (pH 8.0), 150 mM NaCl, 0.5% sodium deoxycholate, 0.1% SDS, 1% NP-40, and 2 mM MgCl_2_], whereas nuclear extracts were prepared according to the Dignam protocol (*67*) containing cOmplete Protease Inhibitor (Millipore, 535140). Both protein extracts were treated with Benzonase (Sigma-Aldrich, E1014-25KU) for 6 hours at 4°C, and the lysate was clarified by centrifugation. Coimmunoprecipitations were performed using FLAG-agarose (Sigma-Aldrich, A2220) or HA-agarose (Sigma-Aldrich, A2095) for overexpression experiments, and IgG (Cell Signaling, 2729S), anti-SIRT2 antibody (Abcam, ab51023), or anti-MOF (Abcam, ab200660) in endogenous immunoprecipitations, by gently rotating at 4°C overnight. The affinity-purified protein complexes were gently washed two times with buffer BC100 and three times with BC500. Complexes were then eluted either using a synthetic peptide (0.4 μg/ml; Sigma-Aldrich, I2149 and F3290) or by acidification with 0.2 M glycine buffer (pH 2.3).

### Subcellular fractionation: Chromatin-free and chromatin-bound fractions

Cellular fractionation to differentiate between chromatin-free and chromatin-bound fractions was performed as previously described (*12*). Briefly, cells were lysed in a buffer containing 1% NP-40, 5 mM MgCl_2_, 10 mM NaCl, and 20 mM tris-HCl (pH 8.0) with protease inhibitors for 30 min on ice, and the lysates were separated by centrifugation (14,000 rpm, 10 min) into supernatant (chromatin-free) and pellet (chromatin-bound) fractions. The pellet was then resuspended in a buffer containing 1% NP-40, 5 mM MgCl_2_, 150 mM NaCl, and 20 mM tris-HCl (pH 8.0) with protease inhibitors and incubated for 30 min on ice followed by sonication.

### Western blotting

Protein extracts and elutions from immunoprecipitations resuspended in Laemmli buffer [2% SDS, 10% glycerol, 60 mM tris-HCl (pH 6.8), and 0.01% bromophenol blue] supplemented with 5% beta-mercaptoethanol were separated by SDS–polyacrylamide gel electrophoresis (PAGE) and transferred to a nitrocellulose membrane (Sigma-Aldrich, GE10600001). Membranes were blocked in 5% skim milk prepared in PBS containing 0.1% Triton X-100. Primary and secondary antibodies were diluted in PBS with 0.2% Tween 20. Antibody incubations were carried out for 1 hour at room temperature, respectively, although, in some cases, primary antibodies were incubated overnight at 4°C. The following primary antibodies were used: anti-Flag (Sigma-Aldrich, 7425), anti-HA (Sigma-Aldrich, 6908), anti-GST (Millipore, 06-332), anti-actin (Sigma-Aldrich, A1978), anti-GAPDH (Invitrogen, AM4300), anti-α-tubulin (Sigma-Aldrich, T5168), anti-Fibrillarin (Santa Cruz Biotechnology, Sc-166001), anti-H3 (Cell Signaling, 9715), anti-H3S10P (Cell Signaling, 9706), anti-H4 (Abcam, ab10158), anti-H4K16ac (Diagenode, C15200219), anti-H4K20me1 (Abcam, ab9051), anti-SIRT2 (Sigma-Aldrich, s8447), anti-MOF (Abcam, ab200660), anti-PHF8 (Abcam, ab36068), anti-PLK1 (Cell Signaling, 4513), anti-CAP-H2 (Abcam, ab200659), and anti-Acetyl-lysine (Cell Signaling, 9814). The following secondary antibodies were used: anti-rabbit HRP (Sigma-Aldrich, A0545) and anti-mouse HRP (Sigma-Aldrich, A9044). Chemiluminescence was carried out using SuperSignal WestDura Extended Duration Substrate (Thermo Fisher Scientific, 34076), and images were captured using an iBright 1500 system (Invitrogen) and analyzed with the Fiji software (*68*).

### Flow cytometry analysis

For cell cycle analysis, cells were trypsinized, washed in PBS containing 2 mM EDTA and 1% FBS, and fixed with 70% ethanol. Fixed cells were then washed and permeabilized in PBS containing 0.5% Triton X-100, 2 mM EDTA, and 1% FBS for 30 min. Cells were stained for 1 hour at room temperature with Alexa Fluor 647 Rat anti-Histone H3 (pS28) antibody (BD Pharmingen, 558609). Last, cells were resuspended in 7-AAD (BD Biosciences, 559925) or propidium iodide (PI; Sigma-Aldrich, P4170), acquired on a FACS Canto II flow cytometer (BD Biosciences) and analyzed with the FlowJo software (BD Biosciences).

For H4K20me1 or CAP-H2 quantification, cells were fixed and permeabilized as described above and then stained with either 1:200 dilution of rabbit anti-H4K20me1 antibody (Diagenode, C15200147) or rabbit anti-CAP-H2 (Abcam, ab200569) for 1 hour at room temperature. Cells were then washed twice with PBS containing 2 mM EDTA and 1% FBS and incubated with secondary anti-rabbit IgG Alexa Fluor 488 (Thermo Fisher Scientific, A-11034) for 1 hour in the dark. Last, cells were resuspended in PI. Samples were acquired on a FACS Canto II flow cytometer and analyzed with the FlowJo software.

For apoptosis assay, we used the Annexin V-FITC Apoptosis Staining/Detection Kit (Abcam, ab14085). Briefly, 10^5^ cells were washed and resuspended in binding buffer in the presence of annexin V–fluorescein isothiocyanate (FITC) and PI. Cells were incubated for 10 min at room temperature in the dark and acquired on a BD FACS Canto II.

For EdU (5-ethynyl-2′-deoxyuridine) incorporation, cells were pulse labeled with 10 mM EdU at 37°C for 45 min and fixed with 70% ethanol. Then, the cells were permeabilized with PBS containing 2 mM EDTA, 1% FBS, and 0.5% Triton for 30 min and stained using the Click-IT EdU Alexa Fluor 647 Imaging Kit (Thermo Fisher Scientific, C10340) with some modifications. Last, cells were resuspended in PI and acquired on a BD FACS Canto II.

### Neutral comet assay

To detect double-strand breaks (DSBs), neutral comet assay was performed using a protocol described previously (*69*). Briefly, cells were embedded in low-melting-point agarose, spread on agarose-coated slides, and lysed overnight. Then, the slides containing the lysed cells were subjected to electrophoresis in neutral tris-borate-EDTA buffer at 11 V for 25 min. The nuclei on slides were stained with Hoechst, and 50 images were processed for each sample using an epifluorescence microscope and analyzed and quantified using the TriTek Comet Score software. The extent of DSB damage was represented by two parameters: tail moment and %DNA in tail.

### Cell viability assay

Cell viability was determined by the MTT (3-(4,5-dimethylthiazol-2-yl)-2,5-diphenyltetrazolium bromide) assay. Briefly, cells were seeded in a 96-well cell culture plate at a density of 3000 cells per well and incubated at 37°C for 24, 48, 72, and 96 hours. After incubation, the medium was discarded and cells were incubated with 100 ml of MTT (Sigma-Aldrich, M2128) 0.5 mg/ml in DMEM with 10% FBS for 4 hours. Last, 100 ml of lysis buffer (16% SDS, 40% dimethylformamide, and 1.2% acetic acid) was added and, after an overnight incubation at 37°C, the OD was measured at 570 nm by a BioTek Synergy H1 Microplate Reader.

### Mass spectrometry

For the identification of Sirt2-dependent deacetylation of MOF, MOF-HA purified was incubated with SIRT2-HA in the presence or absence of NAD^+^ in an in vitro deacetylation assay. Then, eluates were resolved by SDS-PAGE and stained using colloidal Coomassie. Gel bands corresponding to the molecular weight of MOF-HA were excised and subjected to in-gel digestion with trypsin for subsequent MS analysis. The resulting peptides were measured on a Thermo Fisher Scientific Orbitrap Q Exactive Plus coupled to a Thermo Fisher Scientific EASY-nLC with a 60-min reversed-phase gradient in a data-dependent acquisition (DDA) mode. Resulting raw files were analyzed using MaxQuant v2.6.6.0 and a UniProtKB protein database containing the human proteome including unreviewed entries and isoforms. MaxQuant was used with default settings with the following exceptions: (i) Acetyl (K) was selected as additional variable modification, (ii) label-free quantification (LFQ) was enabled, and (iii) intensity-based absolute quantification (iBAQ) was enabled. The resulting output was further analyzed with Perseus v 1.6.14.0.

For MOF interactome analysis, HEK293F cells were transfected with either MOF-WT-HA or MOF-3KR-HA constructs, and the pulled-down complexes were captured using HA-agarose beads. Control cells transfected with an empty vector were included in the experimental design. The immune complexes pulled down from the cells were then subjected to “In-Beads” digestion with trypsin. The resulting peptides mixtures were desalted using C18 stage tips (UltraMicroSpin Column, The Nest Group Inc., MA) and dried down in a SpeedVac. Reconstituted peptides were loaded to 300 μm–by–5 mm C18 PepMap100, 5 mm, 100 Å (Thermo Fisher Scientific) at a flow rate of 15 μl/min using a Thermo Fisher Scientific Dionex Ultimate 3000 chromatographic system (Thermo Fisher Scientific). Peptides were separated using a C18 analytical column (nanoEase M/Z HSS C18 T3 (75 μm by 25 cm, 100 Å, Waters) with a 150-min run, comprising three consecutive steps with linear gradients from 3 to 35% B in 120 min, from 35 to 50% B in 5 min, from 50 to 85% B in 2 min, followed by isocratic elution at 85% B in 5 min and stabilization to initial conditions [A: 0.1% formic acid (FA) in water; B: 0.1% FA in CH_3_CN] at a flow rate of 250 nl/min. The column outlets were directly connected to an Advion TriVersa NanoMate (Advion) fitted on an Orbitrap Fusion Lumos Tribrid mass spectrometer (Thermo Fisher Scientific). The mass spectrometer was operated in a DDA mode. Survey MS scans were acquired in the Orbitrap with the resolution [defined at 200 mass/charge ratio (*m/z*)] set to 120,000. The lock mass was user defined at 445.12 *m/z* in each Orbitrap scan. The top speed (most intense) ions per scan were fragmented by collision-induced dissociation (CID). The tandem mass spectrometry (MS/MS) was detected in the ion trap (with a max injection time of 35 ms). The ion count target value was 400,000 for the survey scan and 10,000 (CID) for the MS/MS scan. Target ions already selected for MS/MS were dynamically excluded for 15 s. The spray voltage in the NanoMate source was set to 1.70 kV. Radio frequency (rf) lens were turned to 30%. Minimal signal required to trigger MS to MS/MS switch was set to 5000, and activation Q was 0.250. The spectrometer was working in positive polarity mode, and singly charge state precursors were rejected for fragmentation. Data were acquired with the Xcalibur software version 4.0.27.10 (Thermo Fisher Scientific). The raw files were analyzed with the MaxQuant (v.1.6.7.0) software using the built-in search engine Andromeda to search against the SwissProt Human database downloaded from the UniProtKB website on 25 October 2019. The search parameters were set as follows: The enzyme was trypsin with a maximum of two allowed missed cleavages. Oxidation in methionines and acetylation at protein N termini were set as variable modifications, whereas carbamidomethylation in cysteines was set as fixed modification. The mass tolerances for the first and main search were set at 20 and 4.5 parts per million (ppm), respectively. In addition, only peptides with more than 8 and up to 25 amino acids were considered. The final list of identified peptides and proteins was filtered by using a 1% false discovery rate (FDR) at both peptide and protein levels. Differential interactor analysis was performed between normalized LFQ protein intensities identified in WT or mutant MOF and matched control samples using a *P* value significance threshold level of 0.05, and the test results were represented as volcano plots. The statistical analysis of the protein-protein interactions was performed with the Significance Analysis of INTeractome (SAINT) algorithm (*70*–*72*).

For PLK1 modifications study, *Wt* and *Kmut* HeLa cells were transfected with FLAG-PLK1 and the transfected protein was immunoprecipitated with anti-FLAG agarose beads. The resulting material was then subjected to “In-Beads” trypsin digestion. Digested peptides obtained from each sample were loaded onto an EvoTip and then separated into an Evosep One (EV-1000) chromatograph system (Evosep) using an EV1137 analytical column (150 μm by 15 cm, 1.5 mm, Evosep) with an 88-min run at a flow rate of 500 nl/min. The column outlets were directly connected to an EASY-IC Ion Source (Thermo Fisher Scientific) fitted on an Orbitrap Eclipse Tribrid mass spectrometer (Thermo Fisher Scientific). The mass spectrometer was operated in a DDA mode. Survey MS scans were acquired in the Orbitrap with the resolution (defined at 200 *m/z*) set to 120,000 covering a mass range of 350 to 1400 *m/z*. The top speed (most intense) ions per scan were fragmented by HCD (higher-energy collisional dissociation). The MS/MS was detected in the Orbitrap (with a max injection time of 54 ms). The ion count target value was 400,000 for the survey scan and 50,000 (HCD) for the MS/MS scan. Target ions already selected for MS/MS were dynamically excluded for 15 s. The spray voltage in the ion source was set to 2.50 kV. rf lens were tuned to 30%. The normalized collision energy for fragmentation was set to 28%. The included charge states for fragmentation were set to 2 to 7. The spectrometer was working in positive polarity mode, and singly charge state precursors were rejected for fragmentation. Data were acquired with the Xcalibur software version 4.2.28.14 (Thermo Fisher Scientific). The raw files were analyzed with the MaxQuant (v.2.3.1.0) software using the built-in search engine Andromeda to search against the SwissProt Human database downloaded from UniProtKB website on 15 March 2023. The search parameters were set as follows: The enzyme was trypsin with a maximum of three allowed missed cleavages. Oxidation in methionines, acetylation at protein N termini, methylation in lysines, and deamidation in asparagines and glutamines were set as variable modifications. Moreover, carbamidomethylation in cysteines was set as fixed modification. The mass tolerances for the first and main search were set at 20 and 4.5 ppm, respectively. In addition, only peptides with more than 8 and up to 25 amino acids were considered. The final list of identified peptides and proteins was filtered by using a 1% FDR both at peptide and protein levels.

### In vitro enzymatic assays

Deacetylation assays with purified SIRT2 and MOF-HA were performed in a reaction buffer containing 50 mM tris-HCl (pH 8.0), 100 mM NaCl, and 2 mM DTT in the presence or absence of 0.5 mM NAD^+^ (Sigma-Aldrich, N8410). MOF-HA was previously purified from HeLa cells treated with 5 μM trichostatin A (TSA) and 5 mM nicotinamide to inhibit endogenous deacetylase activity. Reactions were carried out at 37°C for 2 hours. For Western blot analysis, reactions were stopped by adding Laemmli buffer and acetylation status was assessed using an anti-acetyl-lysine antibody. For MS analysis, reactions were stopped by dialyzing the samples overnight at 4°C in BC100 buffer.

Histone acetylation assays were performed using MOF-HA, and core histones were purified from HeLa cells. Histones were incubated in the presence or absence of MOF-HA in acetylation buffer [50 mM tris-HCl (pH 8.0), 0.1 mM EDTA, 1 mM DTT, and 5% glycerol] supplemented with [^14^C]acetyl–coenzyme A (CoA) for 1 hour at 30°C. Reactions were stopped by the addition of Laemmli buffer, and samples were resolved by SDS-PAGE. Following transfer to a PVDF membrane (Merck), incorporation of [^14^C]acetyl groups into histones was detected by autoradiography. Histone loading was assessed by Coomassie staining, and MOF-HA levels were evaluated by Western blot.

### RNA isolation, cDNA synthesis, and real-time quantitative polymerase chain reactions

Total RNA was isolated from MEFs and HeLa cells using a Maxwell RSC simplyRNA Tissue Kit (Promega, AS1340) according to the manufacturer’s instructions. The cDNA was synthesized from 2 μg of total RNA with a Transcriptor First Strand cDNA Synthesis Kit (Roche, 04379012001) according to the manufacturer’s instructions. Real-time quantitative PCR (RT-qPCR) was performed using the QuantStudio 5 Real-Time PCR System (Thermo Fisher Scientific) with SYBR Green PCR Master Mix (Applied Biosystems, 4312704). Relative gene expression was analyzed in the QuantStudio 5 software (Life Technologies), and values were normalized to the expression of Rpl38, Eeef2, and Rpl19 genes.

### Immunofluorescence analysis

Cells were cultured in poly-d-lysine–coated coverslips until the desired confluence was achieved. Coverslips were washed twice with 1X PBS and incubated for 10 min on ice with preextraction buffer (25 mM Hepes, 50 mM NaCl, 1 mM EDTA, 2 mM MgCl_2_, 300 mM sucrose, and 0.5% Triton X-100). Coverslips were washed twice with 1X PBS and fixed with 4% paraformaldehyde (PFA) (Electron Microscopy Sciences, 15710) for 7 min, followed by a 10-min permeabilization (1X PBS, 1% BSA, and 0.5% Triton X-100) and 1-hour incubation in blocking buffer (1X PBS, 1% BSA, and 0.2% Triton X-100) at room temperature. Primary antibodies were diluted in blocking buffer as follows: mouse anti-H4K20me1 1:200 (Diagenode, C15200147), rabbit anti-H4K16ac 1:200 (Millipore, 07-329), mouse anti-H3S10P 1:500 (Cell Signaling, 9706), and rat anti-H3S28P 1:200 (Sigma-Aldrich, H9908). Incubation with primary antibodies was performed in a humid chamber overnight at 4°C. Next, coverslips were washed three times for 15 min in 1X PBS containing 0.1% Tween 20, and incubated for 1 hour at 37°C with the appropriate secondary antibodies diluted 1:500 in blocking buffer. The following Alexa Fluor–conjugated secondary antibodies were used: Alexa Fluor 555 goat anti-mouse IgG (Thermo Fisher Scientific, A-32727), Alexa Fluor 488 goat anti-rabbit IgG (Thermo Fisher Scientific, A-11034), and Alexa Fluor 488 goat anti-rat IgG (Thermo Fisher Scientificc, A-21208). After three 15-min washes with 1X PBS and 0.1% Tween 20, slides were counterstained with 4′,6-diamidino-2-phenylindole (DAPI) and mounted in Vectashield VIBRANCE mounting media (Vector Laboratories, H-1700). Images were acquired with a Leica Stellaris 8 confocal microscope and analyzed with Fiji.

For the analysis of condensin [CAP-H2 subunit, rabbit anti-NCAPH2 1:100 (Abcam, ab200569)], the permeabilization step was performed before fixation to remove the soluble protein fraction and specifically detect the chromatin-bound pool. After an initial wash with cold PBS, cells were permeabilized in 0.5% Triton X-100 in PBS for 10 min at 4°C, followed by fixation in 3% PFA in PBS for 15 min at room temperature. Subsequent steps were performed according to the general immunofluorescence protocol described above.

### Immunohistochemistry

Immunohistochemistry was performed on 5-μm-thick sections of mouse kidney tissue. Sections were fixed in cold methanol (−20°C), followed by permeabilization in 0.2% Triton X-100 in PBS for 15 min at room temperature. Blocking was carried out in PBS containing 10% normal goat serum (NGS) (Sigma-Aldrich, G9023) and 2% casein (Sigma-Aldrich, C7078) for 1 hour at room temperature in a humidified chamber. Tissues were then incubated overnight at 4°C in a humid chamber with the primary antibody anti-H4K20me1 (Novus Biologicals, 30091) at 1:200 dilution in PBS containing 3% NGS and 2% casein. The next day, sections were washed and incubated for 1 hour at room temperature in the dark with the secondary antibody goat anti-rabbit Alexa Fluor 568, diluted 1:1000 in PBS with 3% NGS and 2% casein. After three washes, nuclei were stained with DAPI (1 μg/ml) for 10 min. Following a final rinse with distilled water, coverslips were mounted using Mowiol mounting medium (Sigma-Aldrich, 81381) containing antifade reagent.

### RNA sequencing

Total RNA was purified from *Wt*, *Sirt2*^−/−^, *Mof*^−/−^, and *Sirt2*^−/−^/*Mof*^−/−^ MEFs exponentially growing using a Maxwell RSC simplyRNA Tissue Kit (Promega) according to the manufacturer’s instructions. After library construction, 50-bp single-end sequencing was performed on an Illumina HiSeq 2500 sequencer. Raw single-end reads were aligned with bowtie2 v2.4.4 to the mouse reference genome (mm39 assembly) (*73*). Uniquely aligned reads overlapping genes were counted with Subread v2.0.3 (featureCounts) (*74*) to obtain the gene count matrix. Next, the DESeq2 package in R was used to calculate differentially expressed genes for the different comparisons shown in this paper (*75*). Gene set enrichment analysis was performed using the GSEA software (v4.3.2) from UC San Diego and the Broad Institute with default settings (add origin of the MMB-FOXM1 gene list) (*76*, *77*).

To identify groups of genes with similar patterns of expression depending on SIRT2 and/or MOF, unsupervised clustering was performed using the R pheatmap package on genes with *P* adjusted < 0.05 after the likelihood-ratio test across all samples. Functional enrichment of cluster genes was performed using the Enrichr platform and the Reactome signature database. To identify regulators involved in SIRT2 transcriptional control, cluster I genes included in the Cell Cycle, Mitotic R-HSA-69278 signature were subjected to transcriptional factor target enrichment analysis using the ENCODE and ChEA Consensus TFs from the ChIP-X database.

Total RNA was purified from *Wt* and *Kmut* HeLa cells exponentially growing using a Maxwell RSC simplyRNA Tissue Kit (Promega) according to the manufacturer’s instructions. After library construction, 150-bp paired-end sequencing was performed on a DNBSEQ-G400 (MGI Tech). Raw single-end reads were aligned with bowtie2 v2.4.4 to the human reference genome (hg38 assembly) (*73*). Uniquely aligned reads overlapping genes were counted with Subread v2.0.3 (featureCounts) to obtain the gene count matrix. Differential expression analysis was performed on the counts matrix using DESeq2. Functional enrichment of up-regulated pathways was performed using Enrichr.

### Chromatin immunoprecipitation

For H4K20me1 ChIP-seq data in MEFs, three confluent 150-cm^2^ dishes of *Wt*, *Sirt2*^−/−^, *Mof*^−/−^, and *Sirt2*^−/−^/*Mof*^−/−^ primary MEF cells were used. First, we cross-linked proteins using 1% of formaldehyde (Electron Microscopy Sciences, 15686), quenched with 125 mM glycine and scraped dishes to get cell pellets. Then, cell lysis was carried out in lysis buffer 1 [5 mM Hepes (pH 7.4), 85 mM KCl, and 0.5% NP-40], and nuclei were collected by centrifugation. Cross-linked chromatin was prepared in lysis buffer 2 [1% SDS, 10 mM EDTA, and 50 mM tris-HCl (pH 8.0)] and fragmented by sonication to an average fragment size of 300 to 500 bp using a Covaris M220 focused ultrasonicator. Then, shearing chromatin was diluted in dilution buffer [1% Triton X-100, 2 mM EDTA, 20 mM tris-HCl (pH 8.0), and 150 mM NaCl] and incubated with primary antibody (H4K20me1; Abcam, ab9051) overnight at 4°C on a rotator. After that, ChIP-grade Protein A/G Magnetic Beads (Thermo Fisher Scientific, 26162) were added to all samples and immunoprecipitated for 6 hours with rotation at 4°C. After fives washes with RIPA buffer [50 mM tris-HCl (pH 8.0), 500 mM LiCl, 1% NP-40, 0.7% sodium deoxycholate, and 1 mM EDTA] and two washes with TE buffer [0.1 mM EDTA and 10 mM tris-HCl (pH 8.0)], DNA elution and reversal of DNA cross-links were performed. Purified DNA using NucleoSpin Gel and PCR Clean-up columns (Macherey-Nagel, 740609) was quantified with the Qubit dsDNA kit (Thermo Fisher Scientific, Q32854), and 10 μg was used for library preparation. Sequencing was carried out on an Illumina HiSeq 2500 sequencing system with a 50-bp singled-end read length.

For H4K20me1 ChIP-seq data in HeLa cells, chromatin was prepared using the truChIP Chromatin Shearing kit (Covaris, 520154) with modifications. Twelve million HeLa cells were harvested and washed once in PBS before fixation in 1% formaldehyde for 10 min. The cross-linking reaction was stopped, and fixed cells were washed twice with ice-cold PBS. Nuclei were purified and sonicated following the manufacturer’s instructions using a Covaris M220 focused ultrasonicator. One volume of 2X dilution buffer supplemented with 0.2% SDS and protease inhibitors was added before clarifying the lysate (10,000*g*, 5 min, 4°C). ChIP was performed overnight at 4°C with 5 μg of an anti-H4K20me1 antibody (Abcam, ab9051). Then, 20 μl of Pierce ChIP-grade Protein A/G Magnetic Beads was added during 3 hours. After immunoprecipitation, samples were serially washed once with low-salt wash buffer [20 mM tris-HCl (pH 8.1), 150 mM NaCl, 0.1% SDS, 1% Triton, and 2 mM EDTA], high-salt wash buffer [20 mM tris-HCl (pH 8.1), 500 mM NaCl, 0.1% SDS, 1% Triton, and 2 mM EDTA], and LiCl wash buffer [10 mM tris-HCl (pH 8), 250 mM LiCl, 1% NP-40, 1% sodium deoxycholate, and 1 mM EDTA] supplemented with protease inhibitors and twice with modified TE buffer [0.1 mM EDTA and 10 mM tris-HCl (pH 8.0)]. Cross-link was reversed in elution buffer (0.1 M NaHCO_3_ and 1% SDS) with 10 μg of RNase A (Thermo Fisher Scientific, EN0531) for 30 min at 37°C followed by an additional 6-hour incubation at 65°C with 50 μg of Proteinase K (Apollo Scientific, BIP4205). DNA was subsequently cleaned up using NucleoSpin Gel and PCR Clean-up columns before library construction and 100-bp paired-end sequencing on a BGISEQ-500.

Raw sequencing reads from ChIP-seq experiments were aligned to the corresponding reference genome (HeLa, human hg38; MEFs, mouse mm39) with bowtie2 v2.4.4 (*73*). Next, unaligned and low-quality alignments were removed with Samtools v1.3 and duplicated reads were removed with Picard Toolkit from Broad Institute (MarkDuplicates) (*78*). DeepTools v3.3.1 was used to generate the bigwig files (bamCoverage) (*79*). Bigwigs were used to compute the signal over the specific regions of interest and plotting (computeMatrix and plotHeatmap, DeepTools v3.3.1).

Public ChIP-seq data for MOF, PHF8, and FOXM1 were obtained from the ENCODE database (ENCSR954KIC, ENCSR604VAE, and ENCSR435ARI, respectively). Peaks were annotated using the R package ChIPseeker to select for peaks bound on genes/promoters (genes bound to intergenic regions were discarded) (*80*).

### Three-dimensional model of human MOF

The model was generated by combining MOF homologous structures from *Mycolicibacterium smegmatis* [Protein Data Bank (PDB) 7CRM, covering MOF residues 175 to 446], from *Mus musculus* (PDB 1WGS, covering MOF residues 55 to 111), and from the human AlphaFold model AF-Q9H7Z6-F1 (covering residues 112 to 174). Regions lacking structural information were modeled using Modeller.

The resulting initial structure was subjected to energy minimization using GROMACS. Structural modifications were then performed using PyMOL. For the K3Ac model, acetylation was introduced at residues K113, K116, and K175. For the 3KR model, the same residues were mutated to arginine (K113R, K116R, and K175R). All modified and mutant models were subsequently energy minimized before structural analysis using PyMOL.

### Breast cancer primary tumors analysis

Matched proteogenomic data from breast cancer primary tumors (*59*) were downloaded from the cBioPortal website. Histone deacetylase including sirtuin protein levels and MOFK113 acetylation levels of primary tumors were downloaded from all samples for which proteomics and acetyl proteomics were available. Low and high SIRT2 or MOFK113ac groups were created according to tumor protein or acetylprotein abundance ratios. Correlations between protein and acetylprotein abundance for each protein were measured using Pearson correlation. To assess the statistical significance of the correlation, a *P* value was also calculated.

### Quantification and statistical analysis

The represented values show means of three or more independent experiments (*n* ≥ 3 with error bars representing the SEM unless otherwise specified). Data were analyzed using a two-tailed Student’s *t* test or one-way analysis of variance (ANOVA) with the Tukey multiple comparisons test (unless otherwise specified), the *P* value was calculated using the GraphPad Prism software.
