# Supplementary material for: SIRT2 antagonizes MOF function during mitotic entry
Source: Sci Adv. 2026 Jun 19;12(25):eaeb2915. doi: 10.1126/sciadv.aeb2915 (PMC13281814; doi:10.1126/sciadv.aeb2915)
Supplement: Supplementary file 1 — Figs. S1 to S6 [file sciadv.aeb2915_sm.pdf]

Science Advances
AAAS

Supplementary Materials for

SIRT2 antagonizes MOF function during mitotic entry

María Espinosa-Alcantud *et al.*

Corresponding author: Alejandro Vaquero, avaquero@carrerasresearch.org

Sci. Adv. **12**, eaeb2915 (2026)
DOI: 10.1126/sciadv.aeb2915

This PDF file includes:

Figs. S1 to S6

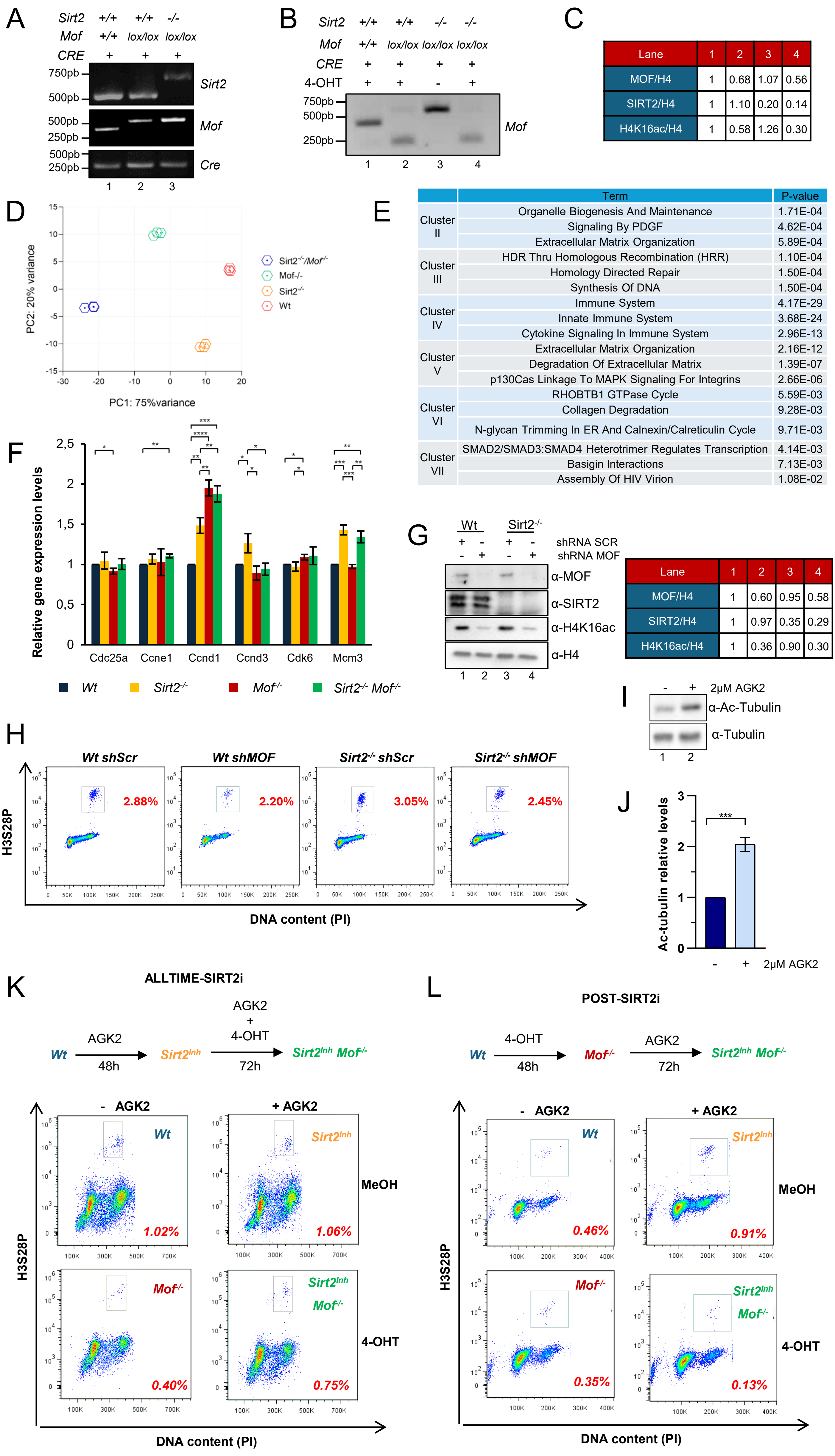

C

Lane	1	2	3	4
MOF/H4	1	0.68	1.07	0.56
SIRT2/H4	1	1.10	0.20	0.14
H4K16ac/H4	1	0.58	1.26	0.30

E

	Term	P-value
Cluster II	Organelle Biogenesis And Maintenance	1.71E-04
	Signaling By PDGF	4.62E-04
	Extracellular Matrix Organization	5.89E-04
Cluster III	HDR Thru Homologous Recombination (HRR)	1.10E-04
	Homology Directed Repair	1.50E-04
	Synthesis Of DNA	1.50E-04
Cluster IV	Immune System	4.17E-29
	Innate Immune System	3.68E-24
	Cytokine Signaling In Immune System	2.96E-13
Cluster V	Extracellular Matrix Organization	2.16E-12
	Degradation Of Extracellular Matrix	1.39E-07
	p130Cas Linkage To MAPK Signaling For Integrins	2.66E-06
Cluster VI	RHOBTB1 GTPase Cycle	5.59E-03
	Collagen Degradation	9.28E-03
	N-glycan Trimming In ER And Calnexin/Calreticulin Cycle	9.71E-03
Cluster VII	SMAD2/SMAD3:SMAD4 Heterotrimer Regulates Transcription	4.14E-03
	Basigin Interactions	7.13E-03
	Assembly Of HIV Virion	1.08E-02

G

Lane	1	2	3	4
MOF/H4	1	0.60	0.95	0.58
SIRT2/H4	1	0.97	0.35	0.29
H4K16ac/H4	1	0.36	0.90	0.30

Figure S1. Generation of SIRT2 and MOF mouse models and their impact on cell cycle. (A) Genotyping of *Sirt2*, *Mof* and *Cre* genes in *Wt* (lane 1), $Mof^{Flox/Flox}$ (lane 2) and $Sirt2^{-/-}$ $Mof^{Flox/Flox}$ (lane 3) mice using genomic DNA extracted from ear punches. **(B)** Genotyping of *Mof* gene in *Wt* (lane 1), $Mof^{Flox/Flox}$ (lane 2), $Sirt2^{-/-}$ $Mof^{Flox/Flox}$ (lane 3 and 4) MEFs treated (lane 1, 2, 4) or not (lane 3) with 4-hydroxytamoxifen (4-OHT). **(C)** Densitometric quantification of MOF, SIRT2 and H4K16ac normalized to H4 levels. **(D)** Principal Component Analysis (PCA) of RNA-seq data from *Wt*, $Mof^{-/-}$, $Sirt2^{-/-}$ and $Sirt2^{-/-}/Mof^{-/-}$ primary MEFs (n=3). **(E)** Reactome analysis of the clusters II to VII of RNA-seq from *Wt*, $Mof^{-/-}$, $Sirt2^{-/-}$ and $Sirt2^{-/-}/Mof^{-/-}$ primary MEFs. The top 3 hits are displayed for each cluster. **(F)** RT-qPCR analysis of G_1/S genes in *Wt*, $Mof^{-/-}$, $Sirt2^{-/-}$ and $Sirt2^{-/-}/Mof^{-/-}$ primary MEFs (mean ± SEM, n=3 independent experiments, one-way ANOVA with Tukey multiple comparisons test). **(G)** MOF, SIRT2 and H4K16ac levels (densitometric quantification normalized to H4 levels is shown) measured by western blot in *Wt* and $Sirt2^{-/-}$ HeLa cells infected with either the scramble or MOF shRNA. Immunoblot for histone H4 as loading control. **(H)** FACS plots of mitotic cells (positive for H3S28P) in *Wt* and $Sirt2^{-/-}$ HeLa cells. DNA content was monitored by PI. In red, the percentage of H3S28P. **(I)** Levels of acetyl-tubulin and total tubulin in immortalized MEFs after no treatment (lane 1) or treatment with 2uM AGK2 (lane 2). **(J)** Quantification of different experiments as in S1H (mean ± SEM, n=6 independent experiments, paired two-tailed t-test). **(K-L)** FACS analysis of mitotic cells (positive for H3S28P) in *Wt* and $Mof^{-/-}$ immortalised MEFs, treated or untreated with 2μM AGK2 before (ALLTIME-SIRT2i) or after (POST-SIRT2i) 4-hydroxytamoxifen treatment. DNA content was stained by PI. $****p < 0.0001$, $***p < 0.001$, $**p < 0.01$, $*p < 0.05$.

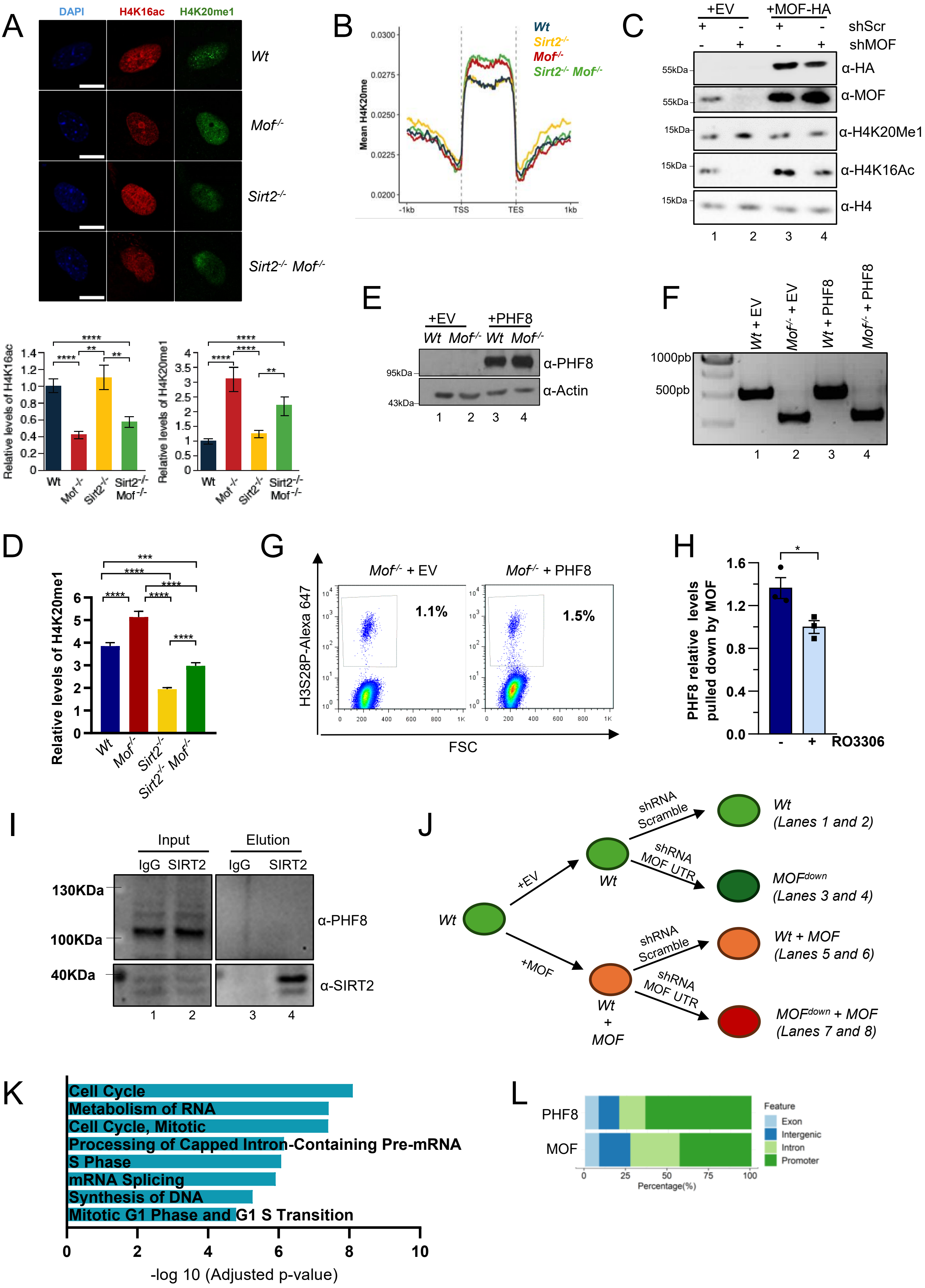
A
DAPI
H4K16ac
H4K20me1
Wt
Mof-/-
Sirt2-/-
Sirt2-/- Mof-/-
Relative levels of H4K16ac
Relative levels of H4K20me1
Wt
Mof-/-
Sirt2-/-
Sirt2-/- Mof-/-
B
Wt
Sirt2-/-
Mof-/-
Sirt2-/- Mof-/-
Mean H4K20me
-1kb
TSS
TES
1kb
C
+EV
+MOF-HA
shScr
shMOF
55kDa
15kDa
α-HA
α-MOF
α-H4K20Me1
α-H4K16Ac
α-H4
E
+EV
+PHF8
Wt Mof-/- Wt Mof-/-
95kDa
43kDa
α-PHF8
α-Actin
F
Wt + EV
Mof-/- + EV
Wt + PHF8
Mof-/- + PHF8
1000pb
500pb
D
Relative levels of H4K20me1
Wt
Mof-/-
Sirt2-/-
Sirt2-/- Mof-/-
G
Mof-/- + EV
Mof-/- + PHF8
H3S28P-Alexa 647
1.1%
1.5%
FSC
H
PHF8 relative levels pulled down by MOF
RO3306
I
Input
Elution
IgG SIRT2 IgG SIRT2
130KDa
100KDa
40KDa
α-PHF8
α-SIRT2
J
Wt
+EV
+MOF
shRNA Scramble
shRNA MOF UTR
Wt
(Lanes 1 and 2)
MOFdown
(Lanes 3 and 4)
Wt + MOF
(Lanes 5 and 6)
MOFdown + MOF
(Lanes 7 and 8)
K
Cell Cycle
Metabolism of RNA
Cell Cycle, Mitotic
Processing of Capped Intron-Containing Pre-mRNA
S Phase
mRNA Splicing
Synthesis of DNA
Mitotic G1 Phase and G1 S Transition
-log 10 (Adjusted p-value)
L
PHF8
MOF
Feature
Exon
Intergenic
Intron
Promoter
Percentage(%)

Figure S2. MOF and SIRT2 play opposite roles in H4K16ac and H4K20me1 dynamics. (A) (Top) Representative H4K16ac and H4K20me1 levels measured by immunofluorescence in *Wt, Mof*$^{-/-}$*, Sirt2*$^{-/-}$ and *Sirt2*$^{-/-}$*/Mof*$^{-/-}$ primary MEFs (Scale bar: 10 μm). (Bottom) Quantification of H4K16ac and H4K20me1 levels measured by immunofluorescence in *Wt, Mof*$^{-/-}$*, Sirt2*$^{-/-}$ and *Sirt2*$^{-/-}$*/Mof*$^{-/-}$ primary MEFs (mean ± SEM, n=2 independent experiments, at least 25 cells analysed per condition, one-way ANOVA with Tukey multiple comparisons test). **(B)** H4K20me1 ChIP-seq signal in annotated genes in *Wt, Mof*$^{-/-}$*, Sirt2*$^{-/-}$ and *Sirt2*$^{-/-}$*/Mof*$^{-/-}$ primary MEFs. **(C)** MOF, H4K20me1 and H4K16ac levels measured by western blot in Wt and MOF Knock-down HeLa cells without (lane 1, 2) or overexpressing MOF-HA (lane 3, 4). Histone H4 blot was used as a loading control. **(D)** Quantification of H4K20me1 levels in kidney cryosections derived from *Wt, Mof*$^{-/-}$*, Sirt2*$^{-/-}$ and *Sirt2*$^{-/-}$*/Mof*$^{-/-}$ mice detected by immunohistochemistry. Scale bar, 50 μm. (mean ± SEM, at least 230 cells analysed per condition, one-way ANOVA with Tukey multiple comparisons test). **(E)** PHF8 levels in *Wt* and *Mof*$^{-/-}$ immortalised MEFs upon PHF8 overexpression. Actin was used as a loading control. **(F)** Genotyping of *Wt* and *Mof*$^{-/-}$ immortalised MEFs upon overexpression of PHF8 after treatment with 4-OHT. **(G)** FACS analysis of mitotic cells (H3S28P positive cells) of *Mof*$^{-/-}$ immortalised MEFs with or without overexpressing PHF8. **(H)** Quantification of PHF8 relative levels to pulled down MOF-HA with or without RO3306 treatment (mean ± SEM, n=3 independent experiments, unpaired two-tailed t-test). **(I)** SIRT2 immunoprecipitation in HeLa cells. PHF8 interaction was assessed by western blot. IgG was used as a negative control. **(J)** Schematic representation of the experiment show in Fig. 2I. **(K)** Reactome of common genes from MOF and PHF8 Chip-seq in HepG2 cells (11867 genes). The top 8 hits are displayed. **(L)** Peak annotation from PHF8 and MOF ChIP-seq from HepG2 cells.

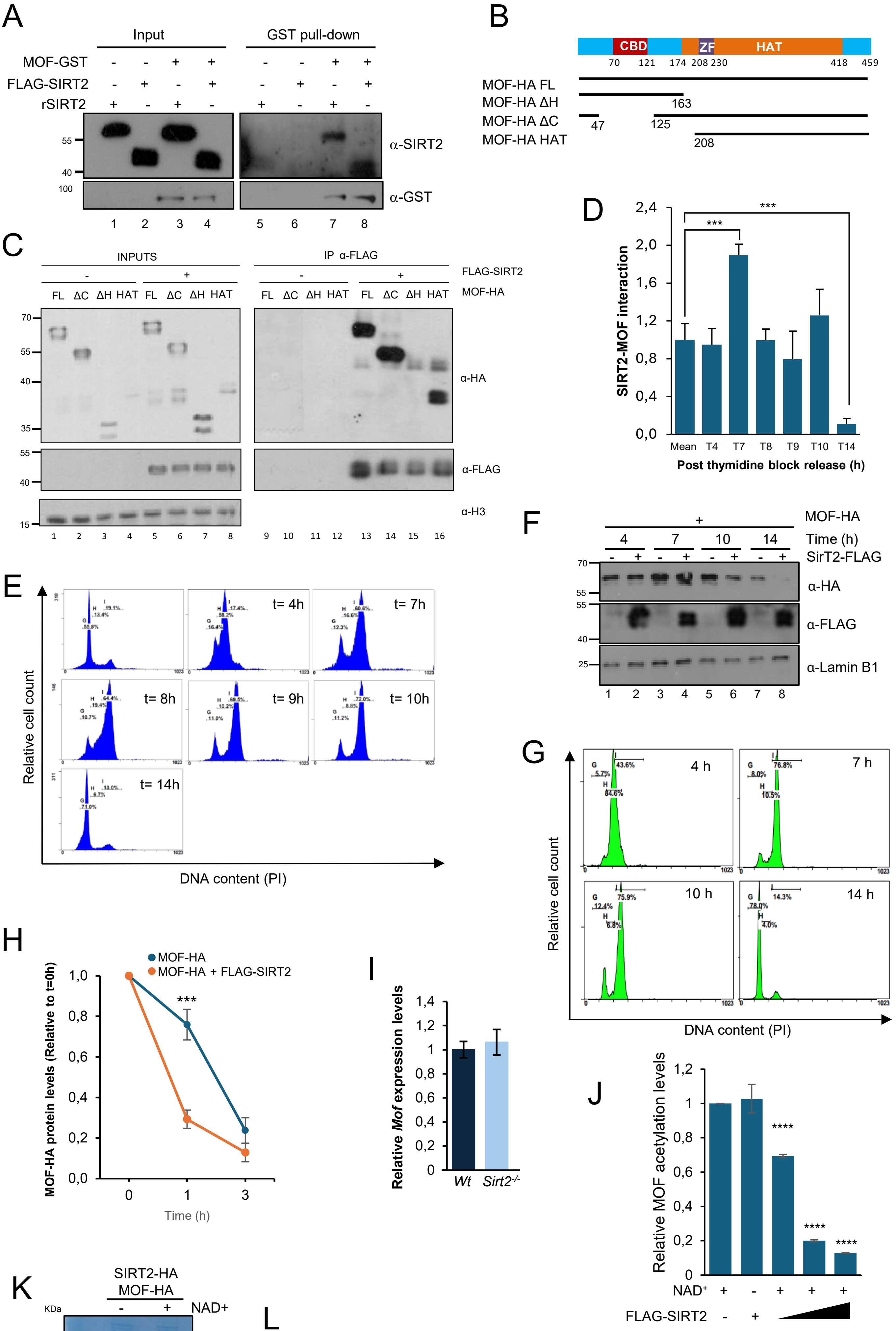

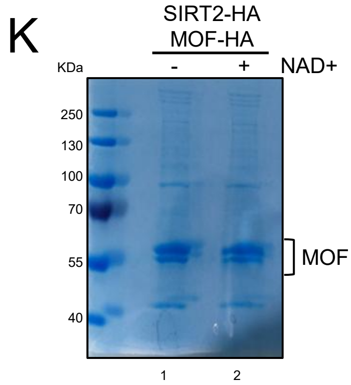

L

MOF	NAD+	Replicates with modification	Localization Probability	Score	PEP
K113ac	-	1/4	1	107,19	0,03
	+	0/4	Not detected	Not detected	Not detected
K116ac	-	3/4	1	203,02	1,07E-07
	+	1/4	1	185,47	9,58E-05
K175ac	-	4/4	1	203,29	1,98E-08
	+	1/4	1	175,51	7,19E-05

Figure S3. SIRT2 interacts with MOF and regulates its activity and stability. (A) Pulldown of MOF-GST performed using human or bacterially expressed SIRT2 (FLAG-SIRT2 or rSIRT2, respectively). **(B)** Schematic representation of the functional domains of hMOF (CBD, Chromo Barrel Domain; ZF, Zinc Finger; HAT, Catalytic acetyltransferase domain) and a diagram of the cloned MOF fragments in comparison to the full-length protein. **(C)** Immunoprecipitation with FLAG resin of FLAG-SIRT2 transfected with the different constructs shown in S2B. **(D)** Bars represent the intensity of co-immunoprecipitated MOF normalized to SIRT2, expressed relative to the mean intensity across all cell cycle time points (mean ± SD, n=3 independent experiments, one-way ANOVA with Dunnett's multiple comparison test). **(E**) Cell cycle profiles of HeLa cells synchronized using double thymidine block obtained by FACS analysis using PI as marked of DNA content. **(F)** Representative western blot of MOF-HA levels in HeLa cells transfected with MOF-HA alone or together with SIRT2-FLAG and synchronized by double thymidine block. Following release from the block, cells were collected at 4, 7, 10, and 14 hours. **(G)** FACS analysis of Hela cells synchronized by double thymidine block and collected at 4, 7, 10, and 14 hours after release. DNA content was monitored by PI. **(H)** Quantification of MOF-HA protein levels after cycloheximide treatment in HeLa cells expressing MOF-HA alone or together with SIRT2 for the indicated times (0, 1 and 3 hours). (mean ± SD, n=3 independent experiments, two-way ANOVA with Tukey's post hoc test). **(I)** Real-time qPCR (RT-qPCR) analysis of MOF levels in *Wt* and *Sirt2*$^{-/-}$ primary MEFs (mean ± SD, n=3 independent experiments, unpaired two-tailed t-test). **(J)** Quantification of MOF acetylation levels normalized to total MOF (mean ± SEM, n=3 independent experiments, one-way ANOVA with Tukey's post hoc test comparing each condition to both controls (−SIRT2/+NAD$^+$ and +SIRT2/−NAD$^+$). **(K)** Colloidal-stained gel of elutions of an in vitro MOF deacetylation assay. **(L)** Localization probability, score and PEP (posterior error probability of the identification) of acetylated sites (K113, K116, K175) of MOF using mass spectrometry analysis. Best values of each modification were reported (all data are available via ProteomeXchange with identifier PXD065214).

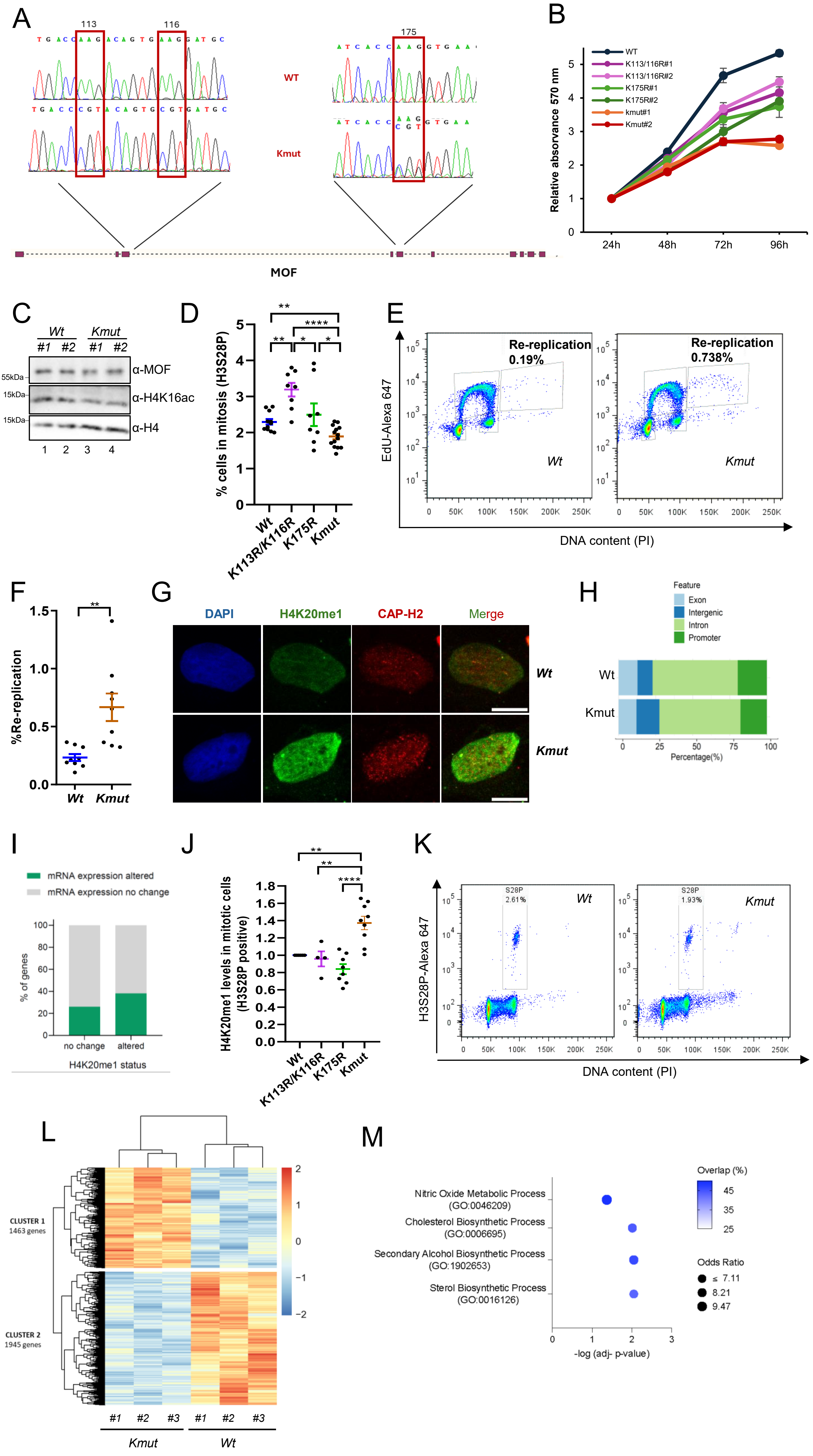
A
113
116
175
WT
Kmut
MOF
B
Relative absorbance 570 nm
WT
K113/116R#1
K113/116R#2
K175R#1
K175R#2
kmut#1
Kmut#2
24h
48h
72h
96h
C
Wt
Kmut
#1 #2 #1 #2
55kDa
15kDa
15kDa
α-MOF
α-H4K16ac
α-H4
1 2 3 4
D
% cells in mitosis (H3S28P)
Wt
K113R/K116R
K175R
Kmut
E
Re-replication 0.19%
Re-replication 0.738%
EdU-Alexa 647
Wt
Kmut
DNA content (PI)
F
%Re-replication
Wt
Kmut
G
DAPI
H4K20me1
CAP-H2
Merge
Wt
Kmut
H
Feature
Exon
Intergenic
Intron
Promoter
Wt
Kmut
Percentage(%)
I
mRNA expression altered
mRNA expression no change
% of genes
no change
altered
H4K20me1 status
J
H4K20me1 levels in mitotic cells (H3S28P positive)
Wt
K113R/K116R
K175R
Kmut
K
S28P 2.61%
S28P 1.93%
Wt
Kmut
H3S28P-Alexa 647
DNA content (PI)
L
CLUSTER 1
1463 genes
CLUSTER 2
1945 genes
#1 #2 #3 #1 #2 #3
Kmut
Wt
M
Nitric Oxide Metabolic Process (GO:0046209)
Cholesterol Biosynthetic Process (GO:0006695)
Secondary Alcohol Biosynthetic Process (GO:1902653)
Sterol Biosynthetic Process (GO:0016126)
-log (adj- p-value)
Overlap (%)
45
35
25
Odds Ratio
≤ 7.11
8.21
9.47

Figure S4. *Kmut* cells recapitulate MOF deficiency in cell cycle and H4K20me1 deposition. (A) CRISPR target loci and Sanger traces showing expected mutations in MOF. The top panel shows the target sequence, and the red box shows the position of the lysines mutated in homozygosis (K113, K116) and heterozygosis (K175). **(B)** MTT assay showing the viability of the different point mutants of MOF obtained by CRISPR/ Cas9 compared to Wt cells (mean ± SEM, n=2 independent experiment using 2 clones per condition, #1 and #2). **(C)** MOF and H4K16ac levels measured by western blot in *Wt* and *Kmut* cells (2 clones tested #1, #2). Immunoblot for histone H4 as loading control. **(D)** Mitotic cells (marked by H3S28P) in *Wt* and the different point mutants of MOF obtained by CRISPR/Cas9 detected by FACS (mean ± SEM, n=3 independent experiments, one-way ANOVA with Tukey multiple comparisons test). **(E)** Cell cycle profiles of *Wt* and *Kmut* HeLa cells obtained by FACS analysis using DNA content (PI staining) in combination with EdU. The values shown are the percentage of polyploid cells (DNA content >4N) with re-replicated DNA. **(F)** Quantification of polyploid cells experiments shown in D (mean ± SEM, n=3 independent experiments, paired two-tailed t-test). **(G)** Levels of H4K20me1 and CAP-H2 in *Wt* and *Kmut* cells determined by immunofluorescence. Scale bar: 10 μm; n = 3. **(H)** Relative genomic distribution of H4K20me1 Chip-seq peaks in *Wt* and *Kmut* cells. **(I)** Relationship between H4K20me1 changes and mRNA expression in *Kmut* vs *Wt* cells. Stacked bar plot showing the proportion of genes with altered or unchanged mRNA expression, stratified by the presence or absence of differential H4K20me1 enrichment. **(J)** H4K20me1 levels in G_2/M measured by FACS in *Wt* cells and the different point mutants of MOF obtained by CRISPR Cas9 (mean ± SEM, n=2 independent experiments, one-way ANOVA with Tukey multiple comparisons test). **(K)** Mitotic cells (marked by H3S28P) in *Wt* and *Kmut* cells detected by FACS. **(L)** Unsupervised clustering of differentially expressed genes (FDR < 0.05) in *Wt* and *Kmut* cells. **(M)** GO enrichment analyses of biological processes of repressed genes in Kmut clones respect *Wt* clones. The top 4 pathways are shown. ∗∗∗∗p < 0.0001, ∗∗p < 0.01, ∗p < 0.05.

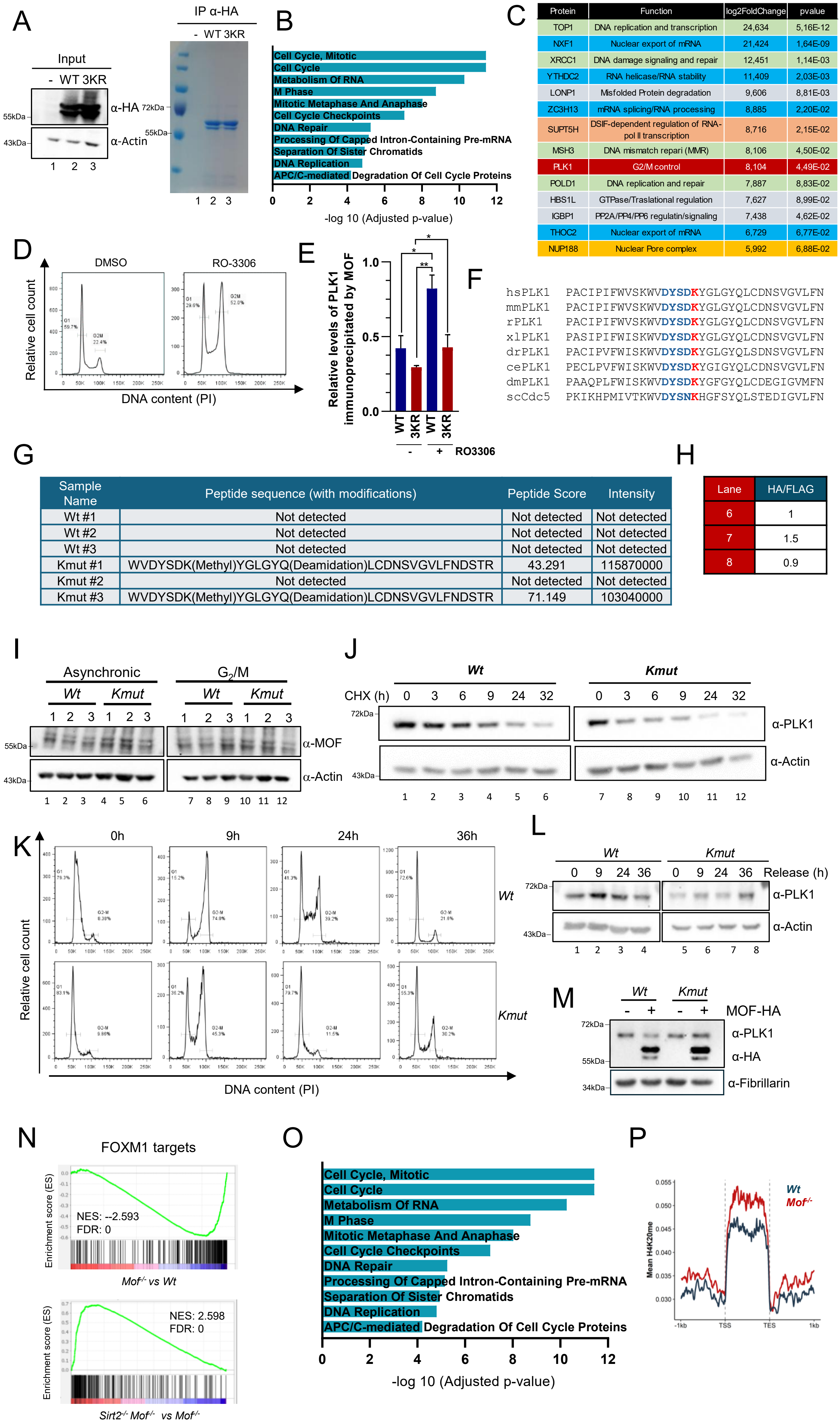

Protein	Function	log2FoldChange	pvalue
TOP1	DNA replication and transcription	24,634	5,16E-12
NXF1	Nuclear export of mRNA	21,424	1,64E-09
XRCC1	DNA damage signaling and repair	12,451	1,14E-03
YTHDC2	RNA helicase/RNA stability	11,409	2,03E-03
LONP1	Misfolded Protein degradation	9,606	8,81E-03
ZC3H13	mRNA splicing/RNA processing	8,885	2,20E-02
SUPT5H	DSIF-dependent regulation of RNA-pol II transcription	8,716	2,15E-02
MSH3	DNA mismatch repari (MMR)	8,106	4,50E-02
PLK1	G2/M control	8,104	4,49E-02
POLD1	DNA replication and repair	7,887	8,83E-02
HBS1L	GTPase/Traslational regulation	7,627	8,99E-02
IGBP1	PP2A/PP4/PP6 regulatin/signaling	7,438	4,62E-02
THOC2	Nuclear export of mRNA	6,729	6,77E-02
NUP188	Nuclear Pore complex	5,992	6,88E-02

Sample Name	Peptide sequence (with modifications)	Peptide Score	Intensity
Wt #1	Not detected	Not detected	Not detected
Wt #2	Not detected	Not detected	Not detected
Wt #3	Not detected	Not detected	Not detected
Kmut #1	WVDYSDK(Methyl)YGLGYQ(Deamidation)LCDNSVGVLFNDSTR	43.291	115870000
Kmut #2	Not detected	Not detected	Not detected
Kmut #3	WVDYSDK(Methyl)YGLGYQ(Deamidation)LCDNSVGVLFNDSTR	71.149	103040000

Lane	HA/FLAG
6	1
7	1.5
8	0.9

Figure S5. MOF/SIRT2 interplay regulates PLK1 protein stability. (A) Representative western blot of MOF-HA-WT and MOF-HA-3KR levels in inputs (left) and pull-downs (right) of the MOF interactors experiment show in Fig 5A. **(B)** Reactome of common interactors of MOF-WT and MOF-3KR. The top 11 pathways are displayed. **(C)** List of MOF-WT specific interactors. Proteins are highlighted in different colors according to their main function: green for DNA repair and damage response, blue for RNA regulation and metabolism, red for cell cycle, yellow for nuclear architecture, and gray for others. **(D)** FACS profile of HeLa cells after treatment with DMSO and RO-3306. **(E)** Quantification of PLK1 relative levels to pulled down MOF-HA-WT and MOF-HA-3KR with or without RO3306 treatment (mean ± SEM, n=3 independent experiments, unpaired two-tailed t-test). **(F)** Alignment of the sequence containing the lysine K420 in the PLK1 lineage. **(G)** Mass spectrometry analysis of PLK1 purified from Wt and *Kmut* cells in G_2/M (3 replicates per condition). The table includes three parameters: peptide sequence, peptide score and intensity. **(H)** Densitometric quantification of Ub-HA normalized to PLK1-FLAG levels. **(I)** Immunoblot of MOF protein levels in asynchronous and G_2/M enriched population in *Wt* and 3 clones of *Kmut* HeLa cells. **(J)** Western blot of PLK1 levels in *Wt* and *Kmut* cells treated with cycloheximide. Actin is used as loading control. **(K)** Cell cycle profiles of *Wt* and *Kmut* HeLa cells synchronized using double thymidine block obtained by FACS analysis using PI as marked of DNA content. **(L)** Immunoblot of PLK1 levels in *Wt* and *Kmut* cells synchronized using double thymidine block. **(M)** Western blot of PLK1 levels in *Wt* and *Kmut* cells without (lane 1, 3) or overexpressing MOF-HA (lane 2, 4). Fibrillarin is used as loading control. **(N)** GSEA plots comparing *Mof*$^{-/-}$ vs *Wt* (top) and *Sirt2*$^{-/-}$/*Mof*$^{-/-}$ vs *Mof*$^{-/-}$ (bottom) are shown. **(O)** Reactome analyses of common proteins show in Fig. 5Q. A selected group of the top 15 hit pathways is displayed. **(P)** H4K20me1 ChIP-seq signal in the genes co-occupied by MOF, PHF8 and FOXM1 in *Wt* and *Mof*$^{-/-}$ primary MEFs.

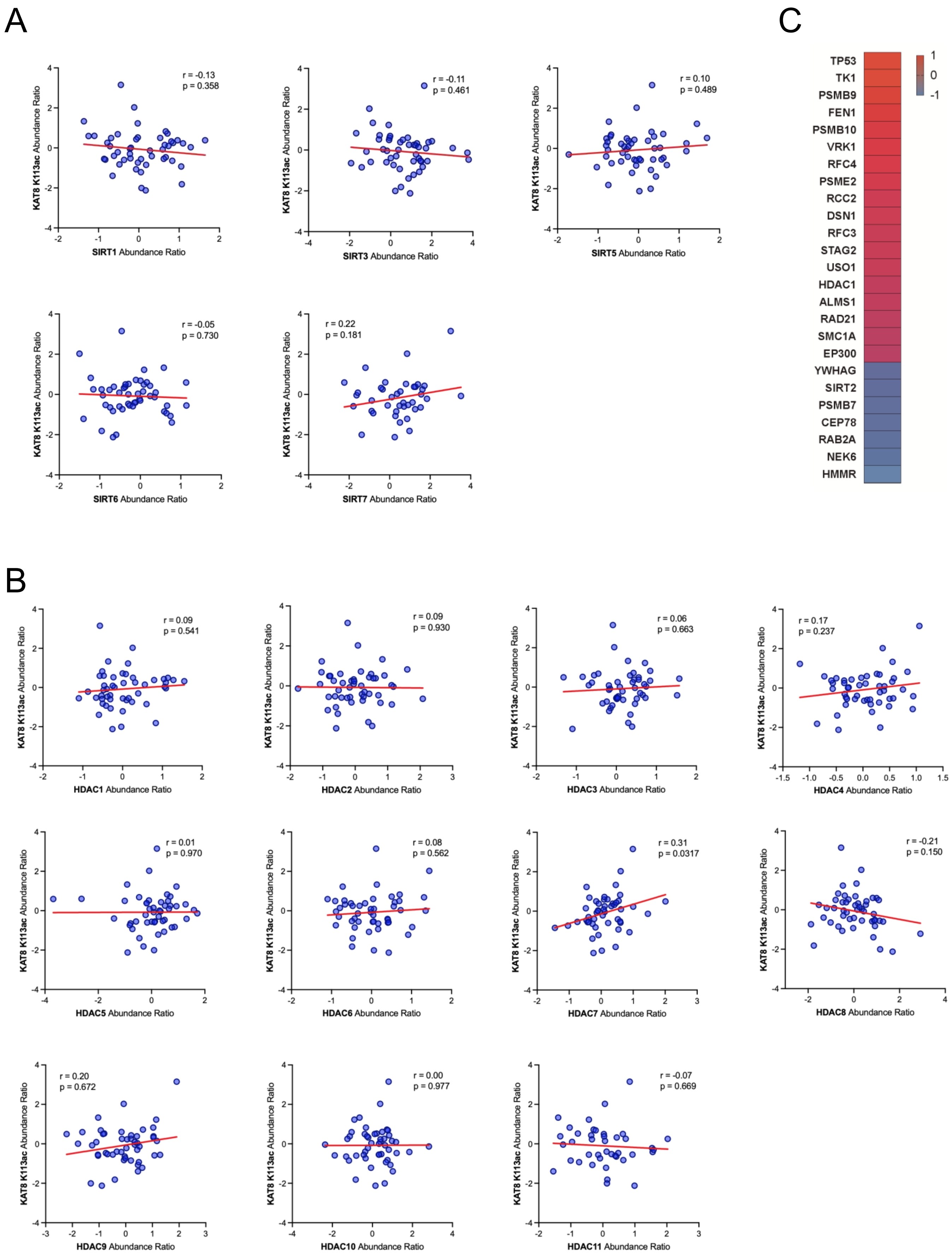
A
C
B
r = -0.13
p = 0.358
KAT8 K113ac Abundance Ratio
SIRT1 Abundance Ratio
r = -0.11
p = 0.461
SIRT3 Abundance Ratio
r = 0.10
p = 0.489
SIRT5 Abundance Ratio
r = -0.05
p = 0.730
SIRT6 Abundance Ratio
r = 0.22
p = 0.181
SIRT7 Abundance Ratio
TP53
TK1
PSMB9
FEN1
PSMB10
VRK1
RFC4
PSME2
RCC2
DSN1
RFC3
STAG2
USO1
HDAC1
ALMS1
RAD21
SMC1A
EP300
YWHAG
SIRT2
PSMB7
CEP78
RAB2A
NEK6
HMMR
1
0
-1
r = 0.09
p = 0.541
HDAC1 Abundance Ratio
r = 0.09
p = 0.930
HDAC2 Abundance Ratio
r = 0.06
p = 0.663
HDAC3 Abundance Ratio
r = 0.17
p = 0.237
HDAC4 Abundance Ratio
r = 0.01
p = 0.970
HDAC5 Abundance Ratio
r = 0.08
p = 0.562
HDAC6 Abundance Ratio
r = 0.31
p = 0.0317
HDAC7 Abundance Ratio
r = -0.21
p = 0.150
HDAC8 Abundance Ratio
r = 0.20
p = 0.672
HDAC9 Abundance Ratio
r = 0.00
p = 0.977
HDAC10 Abundance Ratio
r = -0.07
p = 0.669
HDAC11 Abundance Ratio

Figure S6. SIRT2 is the only HDAC that negatively correlates with MOF acetylation in cancer (A) Correlation plot of MOF acetylation (MOF K113ac) levels and sirtuin protein levels other than SIRT2 (Figure 7E) in primary breast tumours for which both acetylproteomics and proteomics matched data are available (n=49). **(B)** Correlation plot of MOF acetylation (MOF K113ac) levels and HDAC protein levels in primary breast tumours. **(C)** Cell cycle mitotic signature based on differentially expressed proteins between breast cancer tumors with high or low MOF K113ac levels.
